# Review on Carbon Dot-Based Fluorescent Detection of Biothiols

**DOI:** 10.3390/bios13030335

**Published:** 2023-03-02

**Authors:** Muthaiah Shellaiah, Kien Wen Sun

**Affiliations:** Department of Applied Chemistry, National Yang Ming Chiao Tung University, Hsinchu 300, Taiwan

**Keywords:** carbon-dots, biothiols detection, fluorescence, complex-mediated sensors, fluorescence quenching, bioimaging, real analysis, nanocomposites, bioassay, one-pot synthesis

## Abstract

Biothiols, such as cysteine (Cys), homocysteine (Hcy), and glutathione (GSH), play a vital role in gene expression, maintaining redox homeostasis, reducing damages caused by free radicals/toxins, etc. Likewise, abnormal levels of biothiols can lead to severe diseases, such as Alzheimer’s disease (AD), neurotoxicity, hair depigmentation, liver/skin damage, etc. To quantify the biothiols in a biological system, numerous low-toxic probes, such as fluorescent quantum dots, emissive organic probes, composited nanomaterials, etc., have been reported with real-time applications. Among these fluorescent probes, carbon-dots (CDs) have become attractive for biothiols quantification because of advantages of easy synthesis, nano-size, crystalline properties, low-toxicity, and real-time applicability. A CDs-based biothiols assay can be achieved by fluorescent “Turn-On” and “Turn-Off” responses via direct binding, metal complex-mediated detection, composite enhanced interaction, reaction-based reports, and so forth. To date, the availability of a review focused on fluorescent CDs-based biothiols detection with information on recent trends, mechanistic aspects, linear ranges, LODs, and real applications is lacking, which allows us to deliver this comprehensive review. This review delivers valuable information on reported carbon-dots-based biothiols assays, the underlying mechanism, their applications, probe/CDs selection, sensory requirement, merits, limitations, and future scopes.

## 1. Introduction

Detection and quantification of biologically important species are becoming important for treating infections and diseases existing in living systems [1,2,3]. Therefore, bioimaging of these affected tissues or cells was proposed by using fluorescent organic nanoparticles, inorganic nanostructures, hybrid nanosystems, and composites with authenticated evidence [4,5,6,7,8,9,10,11,12,13]. Among these biologically important species, non-protein biothiols, such as cysteine (Cys; normal blood plasma concentration is between 135 to 300 µM), homocysteine (Hcy; normal blood plasma concentration is between 5 to 15 µM), and glutathione (GSH normal blood plasma concentration is between 1 to 6 µM), play a vital role in many pathological process, clinical disorders, and diseases [14,15,16]. Cysteine plays an important role in protein/peptide synthesis, detoxification, cell metabolism, etc., and lack of cysteine may lead to hair depigmentation, liver damage, skin diseases, and cancer [17,18,19]. On the other hand, elevated cysteine levels can cause neurotoxic disorders [20,21]. Subsequently, homocysteine plays a role quite similar to cysteine. However, elevated concentrations of homocysteine in the blood plasma may lead to hyperhomocysteinemia, which is typically categorized into moderate (concentration = 15–30 µM of Hcy), intermediate (concentration = 30–100 µM of Hcy), and severe (concentration ≥ 100 µM of Hcy) disorders [22,23]. In fact, hyperhomocysteinemia can enhance other disorders, such as osteoporosis, dementia, Alzheimer’s disease, cardiac disorders, etc. [24]. Similarly, deficiency in glutathione decreases immunity and enhances the aging process [25]. Elevated levels of glutathione in the human body may enhance the resistance of cancerous cells to chemotherapy [26]. Individual biothiols play important roles in living systems. For example, they can coordinate with biomarkers to afford cancerous cell bioimaging and predict the therapeutic utilities of numerous drug delivery manuals [27,28]. Thereby, detection and quantification of biothiols is a highly important research topic in this field.

To detect and quantify the biothiols, numerous tactics have been proposed, including colorimetric assay, electrochemical methods, fluorescent imaging, surface enhanced Raman spectroscopy, etc. [29,30,31,32]. Among them, fluorescent imaging is rather impressive in terms of the real-time monitoring of biothiols in living tissues or cells [33,34]. Fluorescent sensing of biothiols can be achieved by using organic probes (undergo a reaction with biothiols), functionalized fluorescent quantum dots, hybrid composite nanomaterials, metal-organic frameworks (MOFs), etc. [35,36,37,38,39,40]. Recently, a smartphone-based surface plasmon-coupled emission (SPCE) platform and photonic crystal-coupled emission (PCCE) technology were also employed in biothiol quantification as well as in biosensing studies [41,42,43,44,45,46]. Among these materials, functionalized fluorescent quantum dots have attracted much attention due to their size, photostability, and unique optical properties (Stokes shifts, wide absorption and optimizable PL, etc.) with respect to surface stabilization [47,48]. The easily synthesizable carbon dots (CDs) with a size of <20 nm, which also belong to the quantum dots category, display exceptional opto-electronic properties and have been applied in energetic applications, sensing, bioimaging, therapy, etc. [49,50,51,52,53]. Numerous reports have discussed the detection ability of CDs towards biothiols in cellular imaging and real samples [54,55,56]. In fact, CDs-based detection of biothiols can be achieved by photoinduced electron transfer (PET), intramolecular charge transfer (ICT), Förster resonance energy transfer (FRET), internal filter effect (IFE), aggregation-caused quenching (ACQ), and aggregation-induced emission (AIE), as demonstrated in published works [48,50]. Similarly, fluorescent CDs-based sensing of biothiols can be performed by observing the “Turn-On” and “Turn-Off” florescent responses via the metal ion–CD pair or CDs-based nanocomposites when exposed to biothiols.

**Figure 1:**
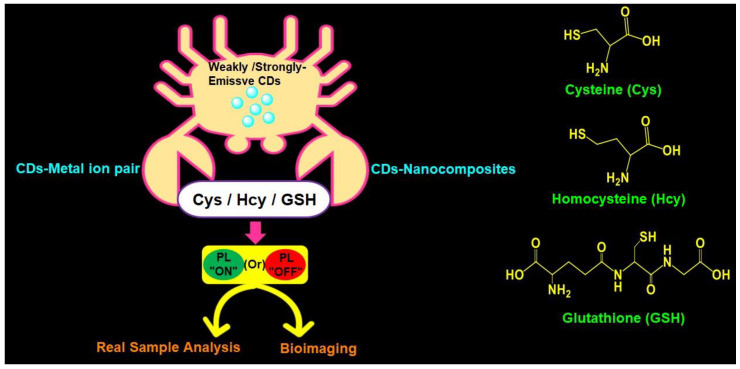
Schematic of fluorescent CDs-based assay of Cys, Hcy, and GSH with applications and structures of Cys, Hcy, and GSH.

Recently, Khan et al. (2020) delivered a comprehensive review covering reports on both CDs and graphene dots (GQDs)-based biothiols sensing [55]. However, to date, the availability of a review focused on fluorescent CDs-based biothiols detection with information on recent trends, mechanistic aspects, linear ranges, LODs, and real applications is lacking, which allows us to deliver this comprehensive review. In this review, the use of emissive CDs in the assay of biothiols (Cys, Hcy, and GSH) is discussed with information on synthesis, photoluminescence quantum yield (PLQY), and demonstrative applications. Moreover, probe/CDs selections, sensory requirements, merits, limitations, and future opportunities for a fluorescent CDs-based biothiols assay are suggested for readers. Figure 1 illustrates schematics of applications and structures of fluorescent CDs-based assay of Cys, Hcy, and GSH.

## 2. Tactics Involved in CDs Synthesis

Before discussing the CDs-based sensory reports for biothiols, this section briefly describes tactics involved in CDs synthesis. Highly emissive CDs can be synthesized by both (A) top-down approaches and (B) bottom-up approaches. Top-down approaches are categorized into (i) arc discharge, (ii) laser ablation, (iii) chemical oxidation, (iv) electrochemical method, and (v) ultrasonic synthesis. Likewise, the bottom-up approaches can be categorized into (i) microwave synthesis, (ii) hydrothermal method, (iii) solvothermal method, (iv) thermal decomposition, and (v) carbonization/pyrolysis method [57,58].

### 2.1. Top-Down Approaches

#### 2.1.1. Arc Discharge

With this method, CDs were synthesized by applying a direct-current arc voltage across two graphite electrodes immersed in an inert gas atmosphere. Chao-Mujica et al. reported synthesis of the fluorescent CQDs using this tactic in water [59]. After purification, these CQDs were consumed in cellular imaging studies; therefore, it was noted as a unique top-down approach.

#### 2.1.2. Laser Ablation

With this method, fluorescent CDs were produced by ablating nanosecond pulse laser over a solid carbon target. The as-synthesized CDs were engaged in cellular imaging studies [60]. Doñate-Buendia et al. synthesized the CQDs with a size of 3 nm via laser irradiation in a continuous flow jet and applied them to cellular imaging studies for prolonged periods of time [61]. In fact, this tactic can produce low toxic CDs for numerous bioimaging/biosensing applications [60,61].

#### 2.1.3. Chemical Oxidation

Chemical oxidation, or exfoliation of a disintegrating bulk carbon source, can be achieved by using a strong oxidizing agent, such as H_2_SO_4_, HNO_3_, NaClO_3_, etc., to produce fluorescent CDs [62]. Desai et al. synthesized fluorescent CDs from muskmelon fruit using sulfuric acid and phosphoric acid as the oxidizing agents [63]. The prepared CDs in the above report were engaged in Hg^2+^ detection and cellular imaging studies, which have motivated researchers to engage in this synthetic tactic.

#### 2.1.4. Electrochemical Method

In the electrochemical method, the oxidation/carbonization takes place by applying an electric field in a chemical environment to produce the fluorescent CDs [64]. This is a rather straight forward method and has been adopted widely in the production of CDs. Lee et al. synthesized fluorescent CDs by the electrochemical method and employed them in the turn-on recognition of chlortetracycline [65], thereby confirming the effectiveness of this tactic.

#### 2.1.5. Ultrasonic Synthesize

In an ultrasonic process, formation and collapsing of small bubbles in liquid produces a strong hydrodynamic shear force to cut the macroscopic carbon materials into nanoscale CDs [66]. Moreover, CDs with diverse properties can be attained by adjusting the ultrasonic power, reaction time, ratio of carbon sources, solvents, etc. Xu et al. developed the multicolour N-doped CDs from kiwi-fruit juice by the ultrasonic synthesis approach and applied these CDs in investigations of fluorescent inks, sensors, and logic gate operations [67].

### 2.2. Bottom-Up Approaches

#### 2.2.1. Microwave Synthesis

By irradiating the electromagnetic wave over the sample at a high temperature, CDs can be produced with exceptional PL quantum yield. In fact, electric dipoles in materials are aligned via microwave-assisted excitation. By optimizing precursor and solvent interactions in the microwave synthesis, CDs with hydrophilic, hydrophobic, or amphiphilic properties can be produced for multiple applications [68]. For instance, Liu et al. demonstrated microwave-assisted synthesis of emissive CDs from citric acid, L-cysteine, and dextrin, and employed them in the real-time detection of Cu^2+^ [69].

#### 2.2.2. Hydrothermal Method

In this method, the reaction mixture in water is placed in a Teflon container and kept in an oven to react hydrothermally at a high pressure and high temperature to produce fluorescent CDs for distinguished applications [57]. For example, Lee et al. synthesized the fluorescent CDs via the hydrothermal method from citric acid, ethylenediamine, and methyl blue and applied them in the “Turn-Off” detection of Hg^2+^ and ClO^−^ [70].

#### 2.2.3. Solvothermal Method

In contrast to the hydrothermal method, the solvothermal tactic replaces the water with one or more organic solvents. The mixtures are sealed with Teflon and subjected to a steel autoclave under a high temperature and high pressure [71]. This method produces highly fluorescent CDs cost-effectively for various applications. Omer et al. discussed the use of solvothermally prepared phosphorous and nitrogen-doped CDs towards Fe^3+^ detection [72], and attested the affordability of the tactic.

#### 2.2.4. Thermal Decomposition

Thermal decomposition (via chemical decomposition) by heating the material or compound was engaged in the production of CDs [73]. This tactic is classified as an endothermic process; however, it was rarely used for CDs synthesis due to its complexity. CDs produced from thermal decomposition were also employed in optoelectronic studies. Wan et al. employed the thermal decomposition tactic to synthesize CDs and graphene-like carbon nanosheets and applied them in optoelectronic device fabrication [74].

#### 2.2.5. Carbonization/Pyrolysis

This is a the most cost-effective, facile, and ultrafast method to synthesize CDs. When organic materials are subjected to prolonged pyrolysis in an inert atmosphere, solid residues with a high carbon content or CDs can be produced with a high yield [75]. Esfandiari et al. synthesized fluorescent CDs by pyrolyzing citric acid in different time periods and temperature ranges. The as-synthesized fluorescent CDs employed in cellular imaging studies showed low toxicity, thereby validating the pyrolysis mediated fluorescent CDs synthesis and suggesting feasible drug delivery applications in the future [76].

### 2.3. Difference between Top-Down and Bottom-Up Approaches

Top-down and Bottom-up approaches can produce CDs with diverse sizes, shapes, and emissive and structural features, as described in Section 2.1 and Section 2.2 and by many reports [77,78,79,80]. Thus, it is essential to summarize their merits and limitations for upcoming researcher. Table 1 summarizes the differences between Top-down and Bottom-up tactics involved in CDs synthesis.

## 3. Fluorescence Mechanism, Importance of PLQY, and Desired Size of CDs

### 3.1. Fluorescence Mechanism of CDs

Synthesized CDs may possess strong, moderate, and weak emission due to the quantum confinement effect, conjugate effect, surface passivation/functionalization effect, surface state, and molecular/carbon-core state properties [81]. Further, the fluorescence of CDs can be tuned via surface passivation, functionalization, doping, and compositing with nanomaterials [82]. In fact, emission of CDs produced by top-down approaches are mostly dependent on surface passivation. On the other hand, bottom-up approaches can produce emissive CDs even without surface passivation [81].

### 3.2. Importance of PLQY of CDs

The PLQY of CDs defines its capability to convert every absorbed photon into fluorescence emission. The PLQY of CDs serves as a correction factor for the determination of multiparameter fluorescence spectroscopy (MFS) parameters, such as FRET, PL quenching efficacy, incorporation of diverse doping/compositing fluorophores and nanomaterials, complex stoichiometry, and decay profiles, etc. [83]. Further, the use of CDs with a higher PLQY affords the high feasibility of long-term bioimaging and tracking of CDs-based drug delivery systems [84]. To achieve CDs with a high PLQY, surface passivation, functionalization, and doping/compositing with nanomaterials can be used, as stated earlier [81].

### 3.3. Desired Size of CDs for Biothiols Quantification

Both Top-down and Bottom-up approaches can produce CDs with a size ranging 1–30 nm [85]. The biothiols assay in real water samples can be performed with emissive CDs with a size ranging 1–30 nm [55]. On the other hand, for the detection and quantification of biothiols in intracellular studies, the size of emissive biocompatible CDs must range 1–10 nm [86]. However, in both cases, the lower the size, the greater the emissive properties of CDs to deliver effective analytical results.

## 4. Representative Mechanism of CDs-Based Fluorescent Biothiols Assay

The CDs-based fluorescent biothiols assay was illustrated by (1) the CD–metal ion pair system [87,88,89,90,91,92,93,94,95,96,97,98,99,100,101,102,103,104,105,106,107,108,109,110,111,112,113,114,115,116,117,118,119,120,121,122,123,124] and (2) CD-nanocomposites [125,126,127,128,129,130,131,132,133,134,135,136,137,138,139,140,141,142,143,144,145,146,147]. Both proposed systems/models deliver the fluorescent response by means of fluorescence recovery or the quenching principle, as clarified next.

### 4.1. Representative PL Mechanism of CD–Metal Ion Pair in the Biothiols Assay

**Figure 2:**
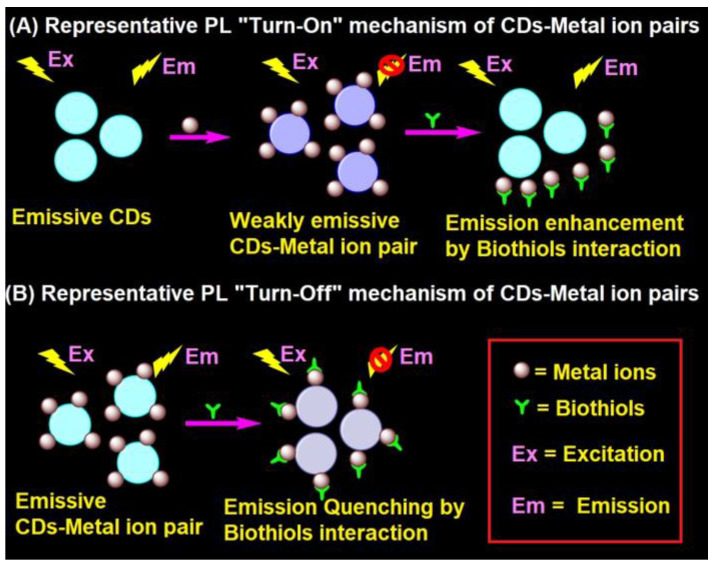
Representative (**A**) PL “Turn-On” and (**B**) PL “Turn-Off” mechanism of the CD–metal ion pair in the biothiols assay.

In general, the interaction of emissive CDs with metal ions (Hg^2+^, Ag^+^, Cu^2+^, Fe^3+^, and Au^3+^) led to fluorescent quenching, which recovers due to the effective interaction of biothiols with metal ions, as shown in Figure 2A [91,92,93,94,95,96,97,98,99,100,101,102,103,104,105,106,107,108,109,110,111,112,113,114,115,116,117,118,120,121,122,123,124]. In fact, the metal ions present in the CD–metal ion pair strongly bind with biothiols via an M^n+^–S interaction to release emissive CDs and exhibit fluorescence recovery. In contrast, CD–metal ion pair with a certain emission may led to fluorescence quenching upon interaction with biothiols, as seen in Figure 2B; however, it has been reported very rarely [119].

### 4.2. Representative PL Mechanism of CD–Nanocomposites in the Biothiols Assay

**Figure 3:**
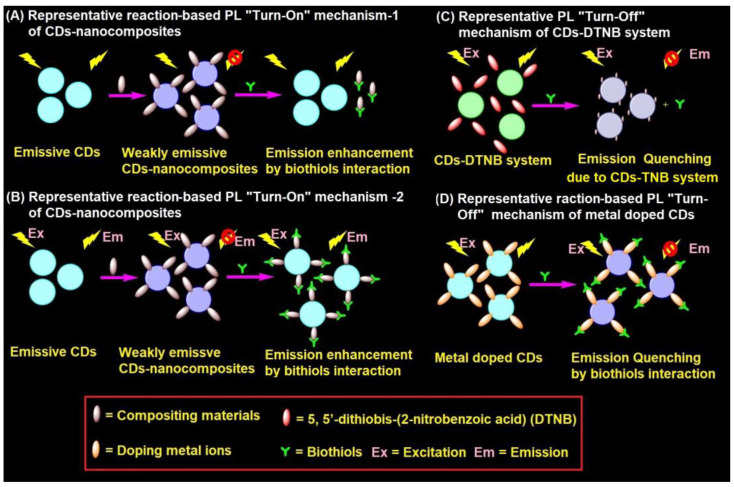
Representative (**A**,**B**) reaction-based PL “Turn-On” mechanism-1 and mechanism-2 of CD–nanocomposites, (**C**) PL “Turn-Off” mechanism of CD–DTNB system, and (**D**) reaction-based PL “Turn-Off” mechanism of metal doped CDs in the biothiols assay.

CD–nanocomposites can be formed by compositing diverse inorganic nanomaterials, conjugation of organic moiety, and doping of metal ions etc., with emissive CDs to afford weakly emissive composites via FRET. These weakly emissive CD–nanocomposites interacts with biothiols to afford two kinds of reaction-based mechanisms, as shown in Figure 3A,B. Biothiols may react with compositing moiety to release emissive CDs (Figure 3A) or interact over the surface of CD–nanocomposites to recovers the fluorescence (Figure 3B). In general, CD–nanocomposites produced by compositing inorganic nanomaterials (such as Au NPs, Ag NPs, and MnO_2_, etc.) and few organic molecule functionalized composite models [125,126,127,128,129,133,134,135,136,137,138,139,140,141] follows the reaction-based mechanism-2 (Figure 3B). On the other hand, few organic moiety functionalized CD–nanocomposite models [142,143,144] follows the reaction-based mechanism-1 (Figure 3A). Subsequently, CD–DTNB (DTNB = 5,5′-dithiobis-(2-nitrobenzoic acid)) dispersed composite model [145,146] has an initial emission due to IFE, which gets disturbed when it interacts with biothiols, resulting in fluorescence quenching, as visualized in Figure 3C. In fact, biothiols react with DTNB and break it into 2-nitro-5-thiobenzoic acid (TNB), which functionalizes over CDs to afford fluorescent quenching (Figure 3C). A rare report on CD–nanocomposites to afford both PL recovery and quenching for discrimination between biothiols via IFE is available [135]. Therefore, the metal ion doped CD–nanocomposite model for biothiols interactive reaction-based fluorescence quenching was also proposed in Figure 3D. However, to date, only cobalt-doped CDs [147] follows the proposed mechanism.

## 5. CD–Metal Ion Pair for Selective Quantification of Biothiols

### 5.1. CD–Hg^2+^ Ion Pair Facilitated Biothiols Assay

Recently, enhanced metal complex-mediated sensing by organic- and colorimetric-nanoprobes has attracted much attention [87,88]. Many CDs-based biothiols assays have been demonstrated via a CD–metal ion pair/complex system. In the beginning, emission of the as-synthesized CDs is quenched by the metal ions via complexation. However, emission can be recovered by exposing to biothiols in solution. During recovery, the affinity between the biothiols and metal ions plays a vital role that leads to the biothiol–metal ion complex formation to restore the CDs original PL emission. In fact, the functional units present in biothiols (such as -SH, NH_2_, -NH, and -COOH) attract metal ions to a certain degree to disrupt the CD–metal complex. For example, the high affinity of Hg to S (present in biothiols) is responsible for disturbing the CD–Hg pairs, which leads to the emission recovery of CDs [89,90]. Therefore, numerous CD-Hg complex systems have been reported for “Turn-On” detection of biothiols [91,92,93,94,95,96,97,98,99,100,101,102,103,104,105,106], as described next.

Zhou et al. synthesized the unmodified blue emissive CDs (CDs; average size = 3.8 nm; λ_em_ = 410 nm at excitation of 340–350 nm; PLQY = 11%) by calcination of di-sodium ethylenediamine tetraacetic acid (EDTA-Na_2_·2H_2_O) at 400 °C for 2 h and was applied in the subsequent detection of Hg^2+^ and biothiols [91]. In the presence of Hg^2+^, the emission peak of CDs at 410 nm was quenched with a decreased PLQY to 8.9% due to the formation of CD–Hg^2+^ pairs with a linear range and a detection limit (LOD) of 0–3 μM (μM = micromole; 10^−6^ M) and 4.2 nM, respectively. When adding biothiols (Cys, Hcy, and GSH), the emission of CDs can be restored via a strong Hg^2+^–S affinity, which extracts Hg^2+^ from the CD–Hg^2+^ complex. The linear regression of Cys, Hcy, and GSH were found as 0.01–5 μM (for all) with a LOD of 4.9 nM (nM = nanomole; 10^−9^ M), 6.1 nM, and 8.5 nM, respectively. This CD–Hg^2+^ complex showed higher selectivity to thiols than that of other amino acid interferences and displayed recoveries of 96.1% and 104.9% in spiked fetal bovine serum (FBS) investigations. This is a unique study, which has initiated similar research in CDs-based biothiols assays.

**Figure 4:**
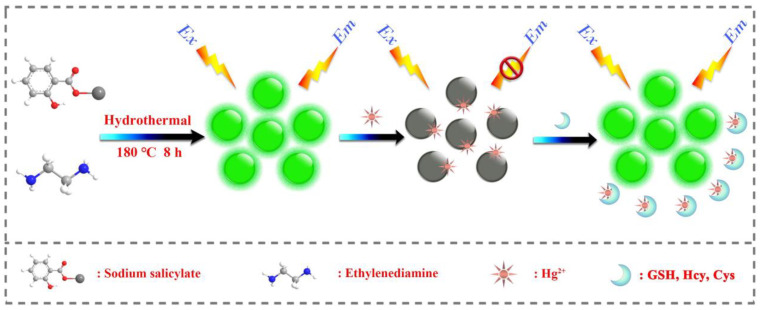
Schematic illustration of the synthesis process of fluorescent CDs and detection principle for Hg^2+^ and biothiols (reproduced with the permission from Ref. [92]).

In a similar approach, green fluorescent CDs (CDs; average size = 2.75–4.25 nm; λ_em_ = 512 nm at excitation of 410 nm; PLQY = 12.3%) were hydrothermally synthesized from sodium salicylate and ethylenediamine (EDA) and were demonstrated in Hg^2+^ complex-mediated sensing of biothiols [92]. The emission of CDs is initially quenched in the presence of Hg^2+^ and then it recovers via interacting with Cys, Hcy, and GSH, as seen in Figure 4. The linear range of Hg^2+^ detection by CDs at pH 6.5 (BR buffer; BR = Briton Robinson buffer (made up from qual mixture of 0.1 M acetic acid, 0.1 M boric acid, and 0.1 M phosphoric acid)) was 0.05–10 μM with a LOD of 44 nM. Subsequently, the linear regression of Cys, Hcy, and GSH was 0.5–10 μM (for all) with LODs of 80 nM, 76 nM, and 69 nM, respectively. This report demonstrated Hg^2+^ and biothiols detection in real water and human plasma samples, respectively, with corresponding recovery of 101.6–105.5% (relative standard deviation (RSD) = 0.31–2.28%) and 91–92.5% (RSD = 0.71–0.99%), hence it can be regarded as an innovative work.

Zhang et al. synthesized the blue emissive CDs (CDs; average size = 4.06 nm; λ_em_ = 440 nm at 360 nm excitation; PLQY = 56%) via the hydrothermal method from N-amino-ethylpiperazine (AEP) and citric acid (CA) and engaged them in the simultaneous detection of Hg^2+^ and biothiols [93], wherein, biothiols detection was implemented through a Hg^2+^ complex-mediated assay tactic. The as-synthesized CDs showed decreasing PL responses between 25 °C and 95 °C. They were also applied in epoxy composite preparation via doping in epoxy resin. In the presence of Hg^2+^, PL emission was quenched linearly (at pH 7.4) at 0–45 µM with a LOD of 90 nM. The emission recovered due to the strong Hg^2+^–S affinity when adding biothiols (Cys, Hcy, and GSH). The Cys, Hcy, and GSH displayed a linear regression at 0–40 µM (for all) with a LODs of 110 nM (for Cys and Hcy) and 130 nM (for GSH). Although this work seems to be innovative, it lacks real-time applications.

Gao et al. synthesized blue emissive CDs (CDs; size = 1.3–2.7 nm; λ_em_ = 450 nm at excitation of 350 nm; PLQY = 81.94%) via the hydrothermal method from folic acid, ammonium citrate, and ethylenediamine and applied them in Hg^2+^ and biothiols detection [94]. This probe displayed dynamic PL quenching to Hg^2+^ linearly from 1–15 µM with a LOD of 80 nM and demonstrated the 85% reversibility with biothiols via a strong Hg^2+^–S affinity. However, details on linear regression, LODs on biothiols detection, and its real-time monitoring are missing.

Xu et al. fabricated the blue emissive nitrogen and sulfur co-doped CDs (NSCDs; average size = 2.7 nm; λ_em_ = 446 nm at excitation of 380 nm) by a one-step pyrolysis from casein and utilized them in sequential discrimination of Hg^2+^ and biothiols (Cys, Hcy, and GSH) [95]. The as-synthesized NSCDs displayed the PLQY of 31.8% with aqueous solubility, photostability, and biocompatibility. At pH 7.4, the PL of NSCDs was quenched in the presence of Hg^2+^ due to NSCDs–Hg^2+^ complexation with a linear range from 0.01–0.25 µM and a LOD of 6.5 nM. This NSCDs–Hg^2+^ complex was disrupted by Cys, Hcy, and GSH, which led to linear recovery of PL emission from 1–10 µM, 0.2–2.5 µM, and 0.1–2.0 µM with LODs of 23.6 nM, 12.3 nM, and 16.8 nM, respectively. In fact, the greater affinity of Hg^2+^–S (from free -SH of biothiols) is the main reason for the PL reversibility. Spiked recovery of Hg^2+^ in water samples and cellular imaging studies of biothiols in HeLa cells (tumor cells) further attested the distinctiveness of this report.

Du and co-workers synthesized the phosphorous containing green emissive CDs (PCDs; average size = 3.2 ± 0.5 nm; λ_em_ = 500 nm at 400 nm excitation; PLQY = 63%) via the hydrothermal method from phytic acid and ethylenediamine and consumed them in sequential detection of Hg^2+^ and biothiols (Cys, Hcy, and GSH) [96]. At pH 7 (10 mM PBS; PBS = (phosphate-buffered saline)), PL emission was quenched linearly from 0–2 µM via PCDs–Hg^2+^ complex formation when adding Hg^2+^ to PCDs. This optimized complex (at fixed 2 µM of Hg^2+^) was disrupted and led to PL recovery due to the strong Hg^2+^–S affinity when adding Cys, Hcy, and GSH. The linear ranges of Cys, Hcy, and GSH were from 0–45 µM, 0–15 µM, and 0–30 µM with LODs of 60 nM, 20 nM, and 35 nM, respectively. This method was also demonstrated in biothiols detection in spiked human urine sample investigations, which showed 95.8–106.2% recovery with 1.6–3.7% RSD values. Thus, this assay tactic can be regarded as innovative.

Pang et al. reported the use of boron and nitrogen co-doped red-emissive CDs (BN-CDs; average size = 2.85 nm; λ_em_ = 616 nm at excitation of 520 nm; PLQY = 18%) towards complex (Hg^2+^/BN-CD)-mediated detection of Cys, Hcy, and GSH [97]. These red-emissive BN-CDs were synthesized from cresyl violet and boric acid via a hydrothermal tactic. Interactions between BN-CDs and Hg^2+^ led to linear fluorescence quenching between 5–175 μM with a LOD of 2.8 μM. When adding Cys, Hcy, and GSH to the above Hg^2+^/BN-CDs system at pH 7.4, fluorescence recovered via a strong Hg^2+^–S bond formation. The linear range of Cys, Hcy, and GSH were 5–200 µM, 5–100 µM, and 5–225 µM with LODs of 1.7 µM, 2.3 µM, and 3 µM, correspondingly. Note that Hg^2+^ detection by red emissive BN-CDs was well attested by spiked real-water interrogations and biothiols-based reversibility was also demonstrated by interference and HepG2 (liver carcinoma cells) cellular imaging studies. However, future work is required to further reduce the LODs.

Blue emissive CDs (λ_em_ = 440 nm at excitation of 350 nm; PLQY = 14.3%) were synthesized by the microwave method and were demonstrated in Hg^2+^–CD complex-mediated “Turn-On” detection of GSH, Cys, and Histidine (His) [98]. This report demonstrated recoverable PL sensing of GSH and Cys with linear ranges of 0.1–20 µM and 0.2–45 µM and LODs of 30 nM and 50 nM, respectively. However, the selectivity could be affected considerably by the presence of His (linear range = 0.5–60 µM; LOD = 150 nM).

Lan et al. synthesized the blue emissive carbon nanoparticles (CNPs; average size = 2.6 nm; λ_em_ = 437 nm at excitation of 350 nm; PLQY = 30%) via the microwave-assisted hydrothermal method from melamine and trisodium citrate dihydrate and engaged them in sequential detection of Hg^2+^ and Cys [99]. When adding Hg^2+^, the PL intensity of CNPs at 437 nm was quenched within 10 s via CNP–Hg^2+^ complex generation. PL intensity recovered when adding Cys to the above complex system (via a strong Hg^2+^–S affinity) with a calculated LOD of 15 nM (linear range = 1–6 µM). The CNP–Hg^2+^ complex probe displayed reversible selectivity to Cys between pH 5 and 10, with a recyclability of more than 10 cycles, and was unaffected in the presence of other interfering analytes. Regarding the biocompatibility of CNPs, detection of Cys in A549 cells (lung tumor cells) attested the real-time imaging utility. Remarkably, the reversibility of the CNP–Hg^2+^ complex was also established with Hcy and GSH, but further investigations are required to determine the linear ranges and LODs. Based on its accomplishments, this report can be regarded as pioneering work.

**Figure 5:**
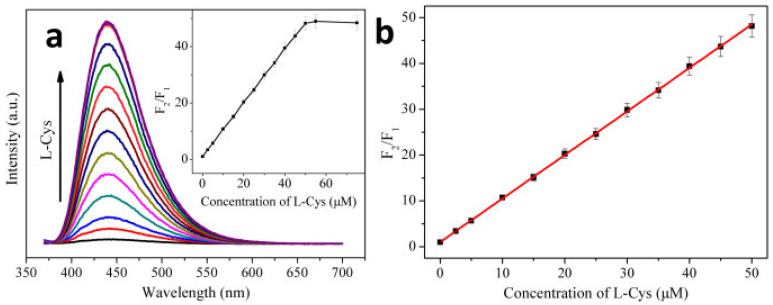
(**a**) PL spectra of the NCD–Hg^2+^ system in the presence of L-Cys with increasing concentrations (from bottom to top: 0, 2.5, 5, 10, 15, 20, 25, 30, 35, 40, 45, 50, 55, and 75 μM). (**b**) A linear relationship of F_2_/F_1_ versus the concentration of L-Cys over the range from 0 to 50 μM. The error bar represents the standard deviation of three measurements (pH 7.0, 0.025 M PBS), λ_ex_ = 360 nm, [NCDs] =16 μg mL^−1^ (μg = microgram, mL = milliliter), [Hg^2+^] = 50 μM; F_1_ and F_2_ are the PL intensities of the NCD–Hg^2+^ system at 440 nm in the absence and presence of L-Cys, respectively) ((**a**,**b**) is reproduced with the permission from Ref. [100]).

Blue emissive nitrogen-doped CDs (NCDs; average size = 2 nm; λ_em_ = 440 nm at excitation of 360 nm; PLQY = 35.4%) were hydrothermally synthesized from citric acid (carbon source) and ammonia solution (nitrogen source) and employed in the consecutive discrimination of Hg^2+^ and L-Cys [100]. PL emission of NCDs at 440 nm was static quenched by Hg^2+^ due to stable NCD–Hg^2+^ complex and was subsequently recovered by adding L-Cys via the greater Hg^2+^–S affinity. The Hg^2+^ complex-mediated quenching showed a linear response of 0–10 μM with a LOD of 1.48 nM, which was also effective in tap water-based interrogations (linear range = 0–10 μM; LOD = 1.65 nM). When adding L-Cys to the NCD–Hg^2+^ complexes at pH 7 (25 mM PBS), PL at 440 nm recovers linearly from 0–50 μM with a LOD of 0.79 nM, as shown in Figure 5. However, similar PL recovery of the NCD–Hg^2+^ complex was not observed when adding DL-Hcy and GSH, thereby confirming the selectivity of L-Cys. This report can be regarded as exceptional, but more focused work on interference and real-time studies is required.

By hydrothermally treating a citron fruit extract and human urine at 180 °C for 7 h, blue emissive nitrogen-doped carbon nanodots (NCNDs; average size = 4 nm; λ_em_ = 455 nm at excitation of 355 nm; PLQY = 34.5%) were synthesized and utilized in sequential “Turn-Off-On” detection of Hg^2+^ and Cys [101]. At pH 7, the fluorescent intensity of NCNDs was quenched in the presence of Hg^2+^ due to NCND–Hg^2+^ complex formation with a linear range of 0–240 µM and a LOD of 150 nM. The NCND–Hg^2+^ complex was disrupted via strong Hg^2+^–S bonds by adding Cys. PL intensity recovered linearly from 1–10 µM with a LOD of 40 nM. The Hg^2+^ and Cys assay by NCNDs was well demonstrated by spiked human urine analysis, which showed recoveries of 90.5–108.8% and 97.08–106.6% with corresponding RSDs of 1.3–2.2% and 1.02–2.06%. Note that NCNDs were successfully used in HeLa cell imaging studies due to low toxicity. Moreover, the selectivity of Hg^2+^ and Cys was not affected by any interferences. In particular, the selectivity of Cys was found to be better than that of Hcy and GSH. Thereby, this work can be noted as innovative.

Tabaraki et al. developed the blue emissive nitrogen and sulfur co-doped CDs (N-S-CDs; diameter size = 30 nm; λ_em_ = 490 nm at excitation of 410 nm) by the microwave assisted hydrothermal synthesis method and applied them in detection of Hg^2+^, Cys, and His [102]. Though this report demonstrated detection of Cys; however, details regarding interreference studies and real-time applications on Cys detection were not available. In fact, the focus of this report was majorly on the fluorescent assay of Hg^2+^ rather than the recovery study.

Zhu et al. clarified the fluorescent-mediated glutathione reductase activity using blue emissive carbon quantum dots (CQDs; average size = 6.5 nm; λ_em_ = 434 nm at excitation of 350 nm) obtained by treating triammonium citrate and disodium phosphate in a microwave-assisted hydrothermal method [103]. The CQDs were complexed with Hg^2+^ to afford a weakly emissive CQD–Hg^2+^ system. By means of glutathione reductase (GR) activity, reduction of oxidized glutathione (GSSG) occurred to release free GSH bound with Hg^2+^ and to restore the PL emission. The linear regression of GR activity was 0.10–2.0 mU mL^−1^ (mU = milli Units) with a LOD of 0.050 mU mL^−1^. This work also demonstrated the lack of GR activity in the presence of the inhibitor 1,3-bis(2-chloroethyl)-1-nitrosourea (BCNU). Likewise, the effect of other enzymes including myoglobin, thrombin, alcohol dehydrogenase, amylase, pepsin, and trypsin on GR activity was also evaluated, thus can be extended towards many biological studies.

Igbal and co-workers synthesized the blue emissive nitrogen-doped CDs (N-CDs; average size = 3 nm; λ_em_ = 440 nm at excitation of 360 nm; PLQY = 40% ± 0.06) via the solid state method (heterogeneously) from anhydrous citric acid and melamine and utilized them in sequential detection of Hg^2+^ and GSH [104]. When adding Hg^2+^ to N-CDs, fluorescence was initially quenched via N-CD–Hg^2+^ complex formation, and thereafter, was restored in the presence of GSH due to a strong Hg^2+^–S affinity. At pH 7.4 (12 mM HEPES; HEPES = (4-(2-hydroxyethyl)-1-piperazineethanesulfonic acid)), the selectivity of N-CDs to Hg^2+^ was excellent compared with other metallic species, which showed linearity at 0–24 µM with a LOD of 20 nM. Likewise, the linear range of GSH was 0–32 µM with a LOD of 40 nM. The GSH also showed better responses than that of Hcy and Cys. The Hg^2+^ and GSH detection by N-CDs was also demonstrated by imaging of BHK-Cells (Baby Hamster Kidney fibroblasts cell line). However, the detail on linear ranges and LODs of Hcy and Cys-based PL recovery of the N-CD–Hg^2+^ complex is unavailable, hence requires further investigations.

Li et al. prepared the blue emissive nitrogen and sulfur co-doped CDs (NSCDs; average size = 4.4 nm; λ_em_ = 430 nm at excitation of 343 nm) from thiomalic acid and urea reacted using the microwave method and applied them in consecutive detection of Hg^2+^ and GSH [105]. At pH 7, the NSCDs complexed with Hg^2+^ and showed linear PL quenching at 0–32 µM with a LOD of 33 nM with negligible interference from other metal ions. PL intensity restored when adding GSH to the NSCD–Hg^2+^ system at a linear concentration of 0.5–34 µM with a LOD of 52 nM. The assay of Hg^2+^ and GSH with this probe was demonstrated by spiked water (for Hg^2+^) and biological samples (human urine and blood serum for GSH) interrogations, which showed recovery of >98% with RSD of <4%. Moreover, HepG2 cellular imaging studies also supported the exceptional use of NSCDs towards Hg^2+^ and GSH quantification. However, this report lacks an interference study on GSH selectivity and sensitivity information on Hcy and Cys, thereby requiring more research on those issues.

**Figure 6:**
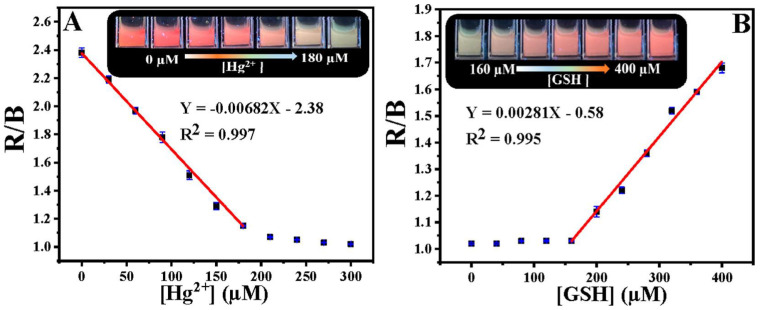
(**A**) R/B values as a function of Hg^2+^ concentrations. Inset: Fluorescent color of NSCDs with different concentrations of Hg^2+^ (0–180 μM) under a 365 nm UV lamp. (**B**) R/B values as a function of GSH concentrations. Inset: Fluorescent color of NSCD–Hg^2+^ with different concentrations of GSH (160–400 μM) under a 365 nm UV lamp ((**A**,**B**) is reproduced with the permission from Ref. [106]).

Chang et al. developed the ratiometric emissive nitrogen and sulfur co-doped CDs (NSCDs; average size = 2.78 nm; λ_em_ = 410 nm and 635 nm at excitation of 360 nm; PLQY = 15.5%) from hydrothermal treatment of 4-aminobenzenesulfonic acid and neutral red and employed them in ratiometric detection of Hg^2+^ and GSH [106]. When adding Hg^2+^ to NSCDs, PL emission at 635 nm was quenched accompanied by enhanced emission at 410 nm due to NSCD–Hg^2+^ complex formation. When exposing the NSCD–Hg^2+^ system to GSH, PL intensity at 635 nm restored ratiometrically accompanied with emission quenching at 410 nm. This is attributed to the disruption of the NSCD–Hg^2+^ complex by a strong Hg^2+^–S affinity induced by GSH. The linear concentration range was established as 0–220 μM for Hg^2+^, 0–200 μM for GSH, and 220–400 μM for GSH with LODs of 7.9 nM and 15.7 nM, correspondingly. Note that the red value and blue value ratio (R/B) displays linear behavior as a function of Hg^2+^ and GSH concentration in the range of 0–180 μM, and 160–400 μM with LODs of 23 nM and 18 nM, respectively, as seen in Figure 6. The NSCDs–Hg^2+^ complex was not affected when adding other interfering amino acids, Hcy, and Cys, thereby displaying a high selectivity to GSH. Moreover, the GSH imaging ability of NSCD–Hg^2+^ was remarkably established in in-vitro and in-vivo imaging studies. Thus, this work can be regarded as innovative with unique biological applications. Table 2 summarizes the linear ranges, LODs, suitable assay pH, and applications of the fluorescent biothiols assay by CD–Hg^2+^ ion pair.

### 5.2. CD–Ag^+^ Ion Pair Directed Biothiols Quantification

Similar to Hg^2+^ complex-mediated detection of biothiols, CDs also interact with Ag^+^ to form a complex, which can be engaged in the reversibility-enabled biothiols quantification, as described next. Shen et al. reported the detection of biothiols via inhibiting the CDs-mediated reduction of Ag^+^ towards the growth of silver nanoparticles (Ag NPs) [107]. The CDs (size = 3.1 ± 1.5 nm) were synthesized solvothermally from chitosan in 2% acetic acid at 180 °C for 12 h. The as-synthesized CDs reduced the Ag^+^ into Ag^0^ to generate Ag NPs, which can be visualized in the absorbance changes at 430 nm (colorimetric changes). This reaction was restricted by Cys, Hcy, and GSH due to a strong Ag^+^–S affinity. The absorbance linear concentrations were found as 2.5–30 nM, 5–40 nM, and 2.5–30 nM with LODs of 20 nM, 2.6 nM, and 1.2 nM, respectively. Although this report demonstrated the biothiols assay in plasma samples, it lacked fluorescence studies.

Biocompatible green emissive CDs (average size = 5 nm; λ_em_ = 530 nm at excitation of 410 nm; PLQY = 32.8% in chloroform and 11.31% in water) were solvothermally prepared from 4-Bromo aniline and ethylenediamine and were engaged in the sequential detection of Ag^+^ and Cys [108]. The as-synthesized CDs were also employed in the detection of triphosgene and latent fingerprint imaging as well. When interacting with Ag^+^, the fluorescent response of CDs at 530 nm was quenched linearly at 0–100 μM with a LOD of 3.9 µM, which can be attributed to CD–Ag^+^ complex formation. When adding Cys to the above system, the emission response was restored via a strong Ag^+^–S bond and the linear concentration was 0–100 μM with a LOD of 3.9 µM. The selective reversibility of the CD–Ag^+^ complex was only demonstrated by Cys, but not by Hcy and GSH. Note that Ag^+^-tuned fluorescent quenching and its reversibility by Cys in aqueous media was well attested by HCT-116 living cell (a human colorectal carcinoma cell line) imaging studies. In terms of multi-analyte detection and latent fingerprint interrogations, this work can be regarded as ground-breaking research. However, further optimization of the LODs and interference studies are still required.

**Figure 7:**
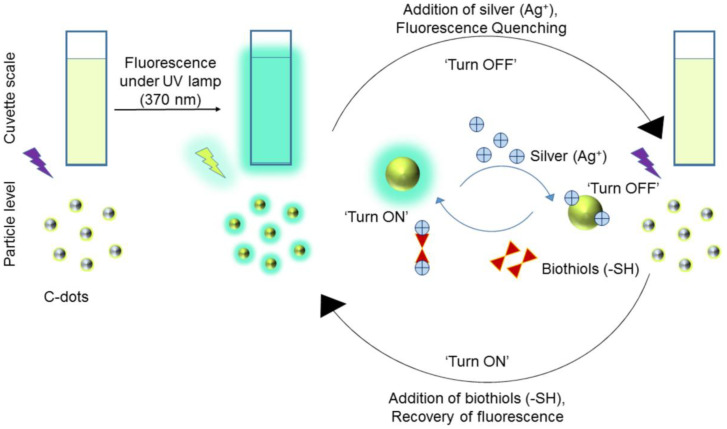
Schematic illustration of the C-dots based ‘turn off-on’ silver-biothiols dual sensing (reproduced with the permission from Ref. [109]).

Borse et al. developed biocompatible blue emissive CDs (average size = 3.3 ± 0.4 nm; λ_em_ = 380 nm at excitation of 320 nm; PLQY = 17.73%) from polyethylene glycol (PEG) by the microwave-assisted hydrothermal method and applied them in consecutive sensing of Ag^+^ and GSH [109]. Because of the CD–Ag^+^ complexation, the fluorescent response was linearly quenched from 1–100 µM with a LOD of 10 nM. When adding GSH to the above system, the fluorescent emission restored via a strong Ag^+^–S bond and the linear range was 1–200 µM with a LOD of 10 nM. The schematic of Ag^+^-enabled fluorescence quenching and biothiols-mediated PL recovery is shown in Figure 7. In fact, the selectivity of Ag^+^ was higher than that of other competing species, but information on interference studies of GSH (also Hcy and Cys) selectivity is insufficient in this report. However, the CDs were also used in the mammalian cell imaging application as supplementary work.

### 5.3. CD–Cu^2+^ Ion Pair Directed Biothiols Discrimination

Towards metal complex-mediated quantification of biothiols, CD–Cu^2+^ complexes were also employed, as described next. Liu et al. synthesized the blue emissive nitrogen-doped carbon quantum dots (PQDs; average size = 4.82 ± 1.82 nm; λ_em_ = 460 nm at excitation of 340 nm; PLQY = 23.2%) from poly(ethyleneimine) by a one-step hydrothermal method and employed them in sequential detection of Cu^2+^ and L-Cys [110]. When adding Cu^2+^ at pH 4 (acetic acid–sodium acetate buffer), PL emission of PQDs was quenched accompanied with a color change from colorless to blue due to PQD–Cu^2+^ complex formation. This complex system was disrupted by L-Cys via a strong Cu^2+^–S bond, which led to PL recovery with a color change from blue to colorless. The linear regression of Cu^2+^ and L-Cys was established as 0–280 μM and 0–800 μM with LODs of 4.75 µM and 4.74 µM (by fluorescence at 460 nm and absorbance at 272 nm, respectively), and 28.11 μM and 19.74 (by fluorescence at 460 nm and absorbance at 272 nm, respectively). This work demonstrated the superior selectivity of L-Cys than that of Hcy and GSH and was also attested by real samples (diluted lemon-flavored beverage (Yantai, China)), tap water (Yantai, China), and logic gate applications. Thus, it can be regarded as innovative but still requires further work towards lowering the LODs.

Red emissive phosphorous- and bromide-doped CDs (P-, Br-CDs; average size = 2–20 nm; λ_em_ = 610 nm at excitation of 450 nm; PLQY = 11.3%) were hydrothermally synthesized from phenylenediamine (pPD), ammonium phosphatedi basic (DAP), and potassium bromide (KBr) and were engaged in the sequential detection of Cu^2+^ and L-Cys [111]. Due to CD–Cu^2+^ complexation, PL emission at 610 nm was linearly quenched at 0–150 μM with a LOD of 4.408 µM. The CD–Cu^2+^ complex system was affected significantly by L-Cys promoted Cu^2+^–S affinity, which led to linear PL recovery at 0–100 μM with a LOD of 2.373 µM. Both Cu^2+^ and L-Cys detection by P- and Br-CDs were attested by real samples with recoveries of >85% and RSDs of <7%. However, information regarding the interference effect on L-Cys selectivity and sensitivity to Hcy and GSH is unavailable.

Guo and co-workers proposed to use blue emissive nitrogen- and sulfur-doped CDs (N, S-CDs; average size = 3.7 nm; λ_em_ = 481 nm at excitation of 380 nm; PLQY = 10%) towards Cu^2+^ complex-mediated detection of Cys, Hcy, and GSH [112]. The N- and S-CDs were hydrothermally synthesized form a mixture of alfalfa and garlic in pure water. The Cu^2+^ complexed system (N-, S-CD–Cu^2+^) displayed weak fluorescence, which delivered a “Turn-On” response to Cys via a strong Cu^2+^–S bond. The linear PL recovery concentration of Cys was found as 0.1–11 µM with a calculated LOD of 86 nM. Note that the human serum sample-based recoveries of Cys ranged from 93.8% to 103.6% with an RSD of 4%. Similar PL recovery of N-, S-CD–Cu^2+^ was also witnessed by Hcy and GSH; however, information on their linear ranges and LODs is unavailable. Nevertheless, in terms of easy synthesis, linear Cys concentrations, and a nanomolar LOD, this report can be regarded as unique work.

**Figure 8:**
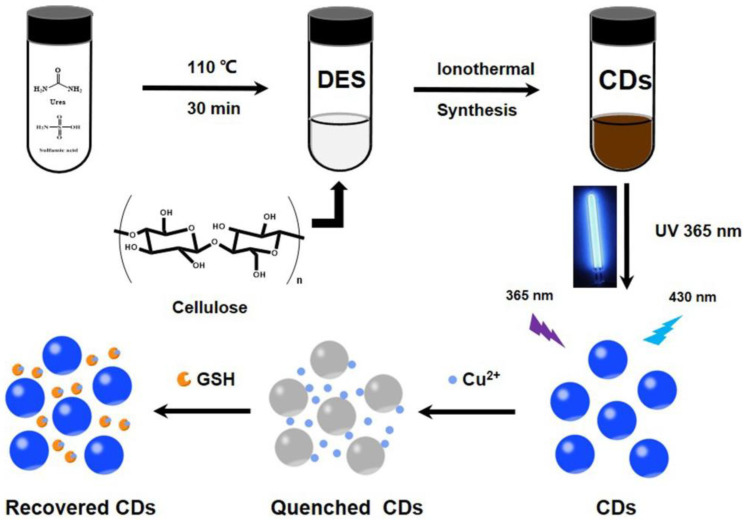
Schematic preparation of N- and S-CDs and their detection for Cu^2+^ and GSH (reproduced with the permission from Ref. [113]).

Wang et al. synthesized blue emissive nitrogen- and sulfur-doped CDs (N, S-CDs; average size = 3.3 nm; λ_em_ = 430 nm at excitation of 360 nm; PLQY = 7.17%) ionothermally from cellulose, urea, and sulfamic acid (deep eutectic solvent (DES) was formed from urea and sulfamic acid) and employed them in sequential discrimination of Cu^2+^ and GSH [113], as shown in Figure 8. When adding Cu^2+^, fluorescence was quenched via the N-, S-CD–Cu^2+^ complex. Emission was recovered through a strong Cu^2+^–S bond when exposed to GSH. Both cases showed higher selectivity than that of other competing species. The linear range of Cu^2+^ and GSH was estimated as 0–1.72 μM and 20–400 μM with LODs of 23.4 nM and 5.98 μM, respectively. Moreover, the GSH selectivity of the N-, S-CD–Cu^2+^ complex was superior than that of Cys. However, this report lacks information of the interference effect and real-time applications.

The blue emissive branched polyethylenimine-functionalized CDs (PEI-CDs; average size = 3.6 nm; λ_em_ = 480 nm at excitation of 365 nm; PLQY = 9.6%) were hydrothermally synthesized from glucose and branched polyethylenimine (PEI) and were engaged in copper complex-mediated detection of GSH [114]. When adding GSH to a weak fluorescent PEI-CD–Cu^2+^ solution, the fluorescent response retained (via a strong Cu–S bond) linearly was 0–80 μM and 0–1400 μM with corresponding LODs of 330 nM and 9.49 µM. Upon incubation of PEI-CD–Cu^2+^ in MGC-803 cells (cell line derived from human gastric carcinoma) with GSH, blue fluorescent cell lines were visualized, which suggested the effectiveness of this approach towards bioimaging studies. The GSH selectivity is superior to that of Hcy and Cys, and is also recyclable for more than five cycles, hence it can be regarded as innovative.

### 5.4. CD–Fe^3+^ Ion Pair Facilitated Biothiols Detection

**Figure 9:**
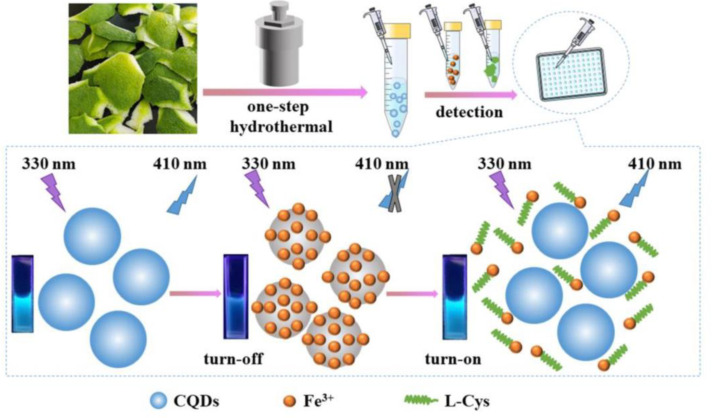
The detection principle for Fe^3+^ and L-Cys based on CQDs (reproduced with the permission from Ref. [115]).

Towards metal complex-prompted detection of Cys, Hcy, and GSH, the CD–Fe^3+^ complex was proposed, as detailed in the following. Zhang et al. prepared the blue emissive CDs (CQDs; average size = 5.5 nm; λ_em_ = 410 nm at excitation of 310–350 nm; PLQY = 17.31%) from green pomelo peel via the one pot hydrothermal synthesis method and employed them in progressive detection of Fe^3+^ and L-Cys, as shown in Figure 9 [115]. The complexation occurred between CQDs and Fe^3+^, which led to PL quenching. The complexation was disrupted by the presence of L-Cys with emission recovery via a strong Fe^3+^–S bond. The linear regressions of Fe^3+^ and L-Cys were 0.1–160 µM and 0.4–85 µM with LODs of 86 nM and 340 nM, respectively. Both the Fe^3+^ and L-Cys assays were supported by recoveries of >80% in water and amino acid beverage interrogations, correspondingly. Moreover, paper-based visual testing strips and cellulose/CQDs composite hydrogel applications on Fe^3+^ and L-Cys selectivity also produced impressive results. However, this work lacks information of the interference effect on Cys selectivity and sensitivity of other thiol containing amino acids (Hcy and GSH), which requires further attention.

Lu et al. synthesized the blue emissive nitrogen-doped carbon nanoparticles (N-CNPs; average size = 62 nm; λ_em_ = 424 nm at excitation of 340 nm; PLQY = 13.1%) from silkworm excrement by the hydrothermal method and employed them in “Off-On” quantification of Fe^3+^ and biothiols (Cys, Hcy, and GSH) [116]. Fluorescence of N-CNPs was initially quenched by adding Fe^3+^ at pH 7 (Tris-HCl = tris-(hydroxymethyl)-aminomethane and hydrochloric acid), and it then recovered by further titration with GSH. This can be attributed to the N-CNP–Fe^3+^ complex and Fe^3+^–S affinity (between GSH and Fe^3+^)-tuned decomplexation. The linear concentration range of Fe^3+^ and GSH was 1–500 µM and 1–1000 µM with LODs of 200 nM and 130 nM, respectively. Detection of GSH was supported by calf-serum based recoveries ranging from 102.4–108.7% with RSDs of <2.5%. Moreover, similar recovery was also observed by Hcy and Cys without linear range and LOD details. However, in terms of green synthesis and simple operation manual, this report can be regarded as distinguished work.

### 5.5. CD–Au^3+^ Ion Pair Aided Biothiols Quantitation

A metal complex-mediated biothiols assay was also demonstrated by the CD–Au complex system, as described subsequently. Gu et al. synthesized the blue emissive carbon dot clusters (CDCs; average size = 3–8 nm; λ_em_ = 440 nm at excitation of 360 nm; PLQY = 7.64%) from black forest honey, deionized water, and ammonia solution by the hydrothermal method. The as-synthesized CDCs were employed in successive discrimination of Au^3+^ and GSH [117]. When adding Au^3+^ to CDCs in solution, PL emission was initially quenched due to CDC–Au^3+^ complex formation, and then recovered with GSH via a strong Au–S bond. The linear concentration ranges of Au^3+^ and GSH were established as 0–75 μM and 0–150 μM with calculated LODs of 0.48 µM and 2.02 µM, respectively. The GSH detection was also demonstrated with biocompatibility of the CDC–Au^3+^ complex in the cellular cytosol study. The selectivity of CDC to Au^3+^ was high. Similar to GSH, Cys, methionine (Met), and dopamine (DA) also displayed PL reversibility to a certain degree, but further optimization is required. Nevertheless, this work initiates research into a CD–Au^3+^ complex-mediated biothiols assay, hence it can be regarded as pioneering research.

Recently, the yellow emissive CDs (YCQDs; average size = 5.3 ± 1.6 nm; λ_em_ = 552 nm at excitation of 410 nm) were hydrothermally synthesized from O-phenylenedimine and ethylene glycol and were utilized in the consecutive detection of Au^3+^ and biothiols [118]. When interacting with Y-CQDs, the Au^3+^ was reduced into Au NPs with an increasing particle size to 11.5 ± 3.5 nm to quench PL emission rapidly. When adding biothiols (Cys, Hcy and GSH) to the above Y-CQDs–Au^3+^ system, PL recovered again due to a strong Au–S affinity. The Au^3+^ shows higher selectivity than that of other metal ions with a linear concentration range of 0–16 µM and a LOD of 59 nM. Likewise, Cys, Hcy, and GSH displayed greater selectivity against other amino acids with linear recovery ranges of 0–20 µM (for Cys and Hcy) and 4–12 µM (for GSH) and LODs of 0.58 µM and 0.62 µM, respectively. The Au^3+^ quantification in mineral water and lake water samples showed recoveries from 99.4% to 105.0% with an RSD of 2.89%. In a similar fashion, a biothiols assay in diluted urine and lemon flavored beverage samples displayed recoveries between 98.4% and 102.5% with an RSD of 2.22%. Moreover, this work was also demonstrated by logic-gate applications. This is the only report using yellow emissive CDs with a demonstrated Au complex-mediated biothiols assay, thereby, it can be regarded as a distinguished work.

### 5.6. CDs-Based “Turn-Off” Detection of As^3+^ and GSH

Gupta et al. synthesized the blue emissive sulfur-doped CDs (CNDs; average size = 4–5 nm; λ_em_ = 425 nm at excitation of 300 nm) from trisodium citrate solution and sodium thiosulphate by microwave-assisted pyrolysis and engaged them in the fluorescent and colorimetric detection of As^3+^ and GSH [119]. PL emission of the CND/GSH system was linearly quenched in the presence of AsO_2_^-^ at 5–50 nM with a LOD of 32 pM (pM = Picomole; 10^−12^ M) due to the GSH/CND–AsO_2_^-^ complex and from the excited state electron transfer process. When adding GSH to CND–AsO_2_^−^, PL was also quenched due to the enhanced effect on electron transfer process at 0–100 nM with a calculated LOD of 43 nM. The selectivity of As^3+^ and GSH was superior to that of other competing species. This is the only report on CNDs-based As^3+^ and GSH detection with the “Turn-Off” response. It can be noted as an initial report with complicated procedures and mechanistic aspects, which requires further attention.

### 5.7. Dye Incorporation in CD–Metal Ion Pair Facilitated Biothiols Assay

Dye molecule-incorporated CDs were employed in a metal complex-mediated ratiometric assay for biothiols, as described next. Wang et al. developed the red emissive CDs (CDs; average size = 2.2–3.7 nm; λ_em_ = 680 nm at excitation of 400 nm) from formamide and glutathione by the microwave-assisted synthesis method. The as-synthesized CDs were then conjugated with fluorescein isothiocyanate (FITC) for sequential ratiometric detection of pH, Ag^+^, and biothiols (Cys, Hcy, and GSH) [120]. PL emission of CDs was quenched with Ag^+^ in a linear concentration range of 1 to 50 µM due to the formation of the CD–Ag^+^ complex and from the dynamic quenching effect (DQE) or static quenching effect (SQE). To achieve the ratiometric fluorescent response with biothiols, FICT conjugated CD–Ag^+^ complexes (CD–F–Ag^+^) were engaged in the PL recovery interrogations. When exposing the CD–F–Ag^+^ complex to Cys, Hcy, and GSH at pH 7.4 (BR buffer), the linear ratiometric PL recovery (at 680 nm/518 nm) was observed as 1.2–4.5 µM, 1.5–6 µM, and 1.2–6 µM with LODs of 55 nM, 68 nM, and 59 nM, respectively. This can be attributed to a strong Ag^+^–S affinity from biothiols. Note that Cys, Hcy, and GSH showed higher selectivity than that of other amino acids and also displayed recovery of >95% in FBS samples with an RSD of <5%. Therefore, it can be regarded as unique research.

Lu et al. synthesized the blue emissive CDs (CDs; average size = 1.5 nm; λ_em_ = 440 nm at excitation of 300 nm) from sodium citrate and histidine by the hydrothermal method. The CDs were then conjugated with rhodamine B (RhB) to be employed in a ratiometric sequential assay of Hg^2+^ and GSH [121]. When adding Hg^2+^ to the CD–RhB nanohybrid system, PL emission at 440 nm/570 nm was linearly quenched between 0.5 and 10 µM with a LOD of 25 nM due to the CD–RhB–Hg^2+^ complex formation. The complex system was disrupted via a strong Hg^2+^–S bond with the incremental addition of GSH accompanied with linear PL recovery (at 440 nm/570 nm) at 0–10 µM with a LOD of 20 nM. The selectivity of Hg^2+^ and GSH was superior to that of other competing species. Similar to GSH, Cys and Hcy also displayed PL reversibility to a certain degree. The GSH assay was also demonstrated by rat serum sample recoveries (>95% with an RSD of <4%). Thus, it is regarded as impressive work.

Fu and co-workers synthesized the blue emissive carbon quantum dots (CQDs; average size = 2–3 nm; λ_em_ = 450 nm at excitation of 380 nm; PLQY = 32%) from sodium alginate and histidine by the hydrothermal method. The CQDs were conjugated with RhB to be consumed in ratiometric sequential assay of Hg^2+^ and GSH [122]. Subsequent titrations of Hg^2+^ and GSH with CQD–RhB led to CQD–RhB–Hg^2+^ complex formation and Hg^2+^–S affinity tuned ratiometric PL quenching and recovery (at 450 nm/570 nm). The linear regressions of Hg^2+^ and GSH quantification were 0.1–40 μM and 0.08–60 µM with LODs of 30 nM and 20 nM, correspondingly. Similar to the GSH-enabled PL recovery, Cys and Hcy also displayed PL reversibility at higher concentrations. The selectivity of both Hg^2+^ and GSH was superior as demonstrated in spiked recovery studies in water (for Hg^2+^) and food (for GSH) samples. Hence, it can be regarded as remarkable sensory research. Based on previous reports [91,92,93,94,95,96,97,98,99,100,101,102,103,104,105,106,107,108,109,110,111,112,113,114,115,116,117,118,119,120,121,122], it can be concluded that CD-metal ion pairs can act as a unique sensory array towards multi-channel fluorescent assays of biothiols [123,124].

## 6. CD Incorporated Nanocomposites for Biothiols Detection

### 6.1. Au@CD Nanobeacons Directed Biothiols Assay

Towards the selective quantification of biothiols, CD incorporated composites plays a vital role, as detailed in this section. Mandani et al. synthesized the blue emissive CDs (CDs; average size = 3.5 ± 0.8 nm; λ_em_ = 470 nm at excitation of 370 nm) by using microwave pyrolysis of β-carotene in water followed by compositing with Au NPs to afford weakly emissive Au@C-dot nanobeacons, and engaged them in the “Turn-On” detection of biothiols [125]. The occurrence of FRET between Au NPs and CDs is responsible for the observed dynamic fluorescence quenching, which was disrupted by ligand exchange via the Au–S bond of free sulfhydryl groups existing in biothiols. When adding Cys to the Au@C-dot system, the fluorescence recovered linearly from 0–30 μM with a LOD of 50 nM. Similar PL reversibility was also observed with GSH, penicillamine (Pen; a thiol containing drug), and thiol containing protein and enzymes (namely bovine serum albumin (BSA), urease, pepsin, glucose oxidase (GOx), urease), hence this can be regarded as a unique tactic towards a biothiols assay. However, much attention is required towards scrutinizing the interference effect.

### 6.2. MnO_2_@CQD Aided Biothiols Detection

Garg and co-workers synthesized the blue emissive carbon quantum dots (CQDs; average size = 7 nm; λ_em_ = 420 nm at excitation of 320 nm) from ascorbic acid and kollicoat by the microwave method followed by combining with manganese dioxide to afford weakly emissive MnO_2_@CQD nanocomposites, and employed them in the “Turn-On” detection of biothiols (including Cys, GSH, 6-thioguanine (6-TG) and 6-mercaptopurine (6-MP), and enapril (an anti-cancer drug)) with nanomolar LODs [126]. This report also described similar mechanistic approaches (FRET and ligand exchange via Mn–S bond), but lacks details on interference studies.

### 6.3. CD-Nanocomposites for Reaction-Based Quantification of Biothiols

Sun et al. synthesized the green emissive CDs (g-CDs; average size = 2.94 nm; λ_em_ = 505 nm at excitation of 407 nm; PLQY = 23%) by the one-step solvothermal treatment of 3-diethylaminophenol followed by functionalizing with 2,4-dinitrobenzenesulfonate to afford weakly emissive g–CD–DNBS (a composite model) and engaged them in the “Turn-On” detection of biothiols (Cys, Hcy, and GSH) [127]. In the presence of biothiols (in phosphate buffer; pH 7.4, 20 mM (mM = millimole; 10^−3^ M), 10% acetone), the functionalized DNBS cleaved and reacted with biothiols to release the highly emissive g-CDs (maximum intensity attained within 30 min). The probe (g–CD–DNBS) shows higher selectivity to Cys, Hcy, and GSH than that of other competing species. Linear regression of Cys, Hcy, and GSH were 0.2–10 µM (for Cys and GSH) and 0.2–12 µM (for Hcy) with corresponding LODs of 69 nM and 74 nM, respectively. This work was also demonstrated in FBS samples and SMMC-7721 cellular (hepatocellular carcinoma cell line) imaging studies, hence it can be regarded as advanced research.

**Figure 10:**
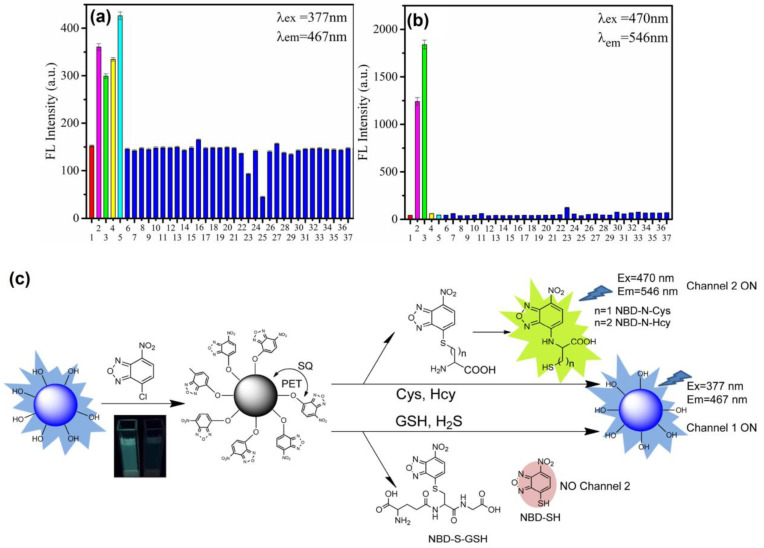
(**a**,**b**) Fluorescence responses of the CQD–O–NBD solution (10 μg mL^−1^) to biothiols (30 μM) and some relevant analytes (100 μM): (1) none, (2) Cys, (3) Hcy, (4) GSH, (5) NaHS, (6) Arg, (7) Asp, (8) Glu, (9) His, (10) Lys, (11) Ser, (12) Thr, (13) Trp, (14) Tyr, (15) Val, (16) Gly, (17) Phe, (18) Ala, (19) Pro, (20) Iso, (21) Leu, (22) Br^−^, (23) CO_2_^3−^, (24) HCO_3_^−^, (25) ClO^−^, (26) Ca^2+^, (27) Fe^3+^, (28) Mg^2+^, (29) Pb^2+^, (30) Zn^2+^, (31) K^+^, (32) Na^+^, (33) NO_3_^−^, (34) PO_4_^3−^, (35) I^−^, (36) CH_3_COO^−^, and (37) Vitamin C. All experiments were carried out in ethanol/PBS and aqueous solution (vethanol/vPBS = 1/1, CPBS = 10 mM and pH = 7.4). Each spectrum was measured for 120 min (λ_ex_ = 377 nm) and 60 min (λ_ex_ = 470 nm) after the analytes were added. Error bars represent ± SD of three experiments (**c**) The proposed reaction mechanism of CQD–O–NBD with Cys/Hcy and GSH/NaHS ((**a**–**c**) is reproduced with the permission from Ref. [128]).

Thereafter, blue emissive carbon quantum dots (CQDs-OH synthesized via the microwave method; average size = 5.20 ± 0.93 nm; λ_em_ = 467 nm at excitation of 377 nm; PLQY = 19.3%) were conjugated with 4-chloro-7-nitrobenzo-2-oxa-1,3-diazole (NBD-Cl) to afford weakly fluorescent CQD–O–NBD (a composite model) and were engaged in dual channel discrimination of Cys/Hcy and GSH/H_2_S [128]. The weak fluorescent nature of CQD–O–NBD was attributed to the static quenching (SQ) and the photo-induced electron transfer (PET) from CQDs to NBD. At pH 7.4 (10 mM phosphate buffer), the presence of thiol compounds, such as Cys, Hcy, GSH, and NaHS (NaHS = sodium hydrosulfide (source for H_2_S generation)), triggered the nucleophilic reaction to cleave CQD–O–NBD and to release CQD–OH, which can be visualized by a “Turn-On” response at 467 nm (excited at 377 nm). The above process was denoted as channel 1. When exciting at 470 nm, the PL “Turn-On” response at 546 nm was triggered by Cys and Hcy instead of GSH and NaHS, which was denoted as channel 2. Due to the steric effect, the intramolecular re-arrangement is restricted in GSH, thus no green channel emission can be observed. The responses from both channels show higher selectivity than that of other competing species due to the nucleophilic reaction, as seen in Figure 10. The linear ranges of Cys, Hcy, GSH, and NaHS in the channel 1 response were established as 0.5–15 μM, 0.5–10 μM, 0.5–12.5 μM, and 0–10 μM with calculated LODs of 0.24 µM, 0.210 µM, 0.11 µM, and 0.18 µM, respectively. Likewise, the linear ranges of Cys and Hcy in the channel 2 response were 0.5–15 μM and 0.5–25 μM with estimated LODs of 70 nM and 60 nM, correspondingly. This dual channel discrimination between Cys/Hcy and GSH/H_2_S was also attested by HeLa cellular imaging studies and in FBS samples, which showed recoveries of 99.7–103.2%. This is a unique work to be utilized for real-time discrimination between Cys/Hcy and GSH/H_2_S.

Ortiz-Gomez et al. developed a vinyl sulfone clicked carbon dot-engineered microfluidic paper-based analytical device towards the fluorometric detection of Cys, Hcy, and GSH [129]. In this report, the free amine containing CDs (CDs; average size = 3 nm; λ_em_ = 450 nm at excitation of 330 nm) were synthesized from poly-ethyleneimine by the hydrothermal treatment at 180 °C for 10 h. The divinyl sulfone (DVS) was anchored on a Whatman grade-1 filter paper to afford VS-paper disks, which interacted with CDs (containing free amines) via the click reaction to deliver fluorescent microfluidic paper-based analytical device (μPAD; CDs-cellulose nanocomposites). The μPAD reacted with iodoacetic acid (IAA) (via free amine of CDs) to afford non-fluorescent CD-I paper. The PL response can be recovered in the presence of Cys, Hcy, and GSH. The linear regressions of Cys, Hcy, and GSH were 5–200 μM (for Cys and Hcy) and 1–200 µM (for GSH) with LODs of 0.3 µM (for Cys and GSH) and 0.4 µM (for Hcy), respectively. The microfluidic paper-based analytical device shows higher selectivity than that of other competing amino acids and also showed recoveries of >98% in urine sample analysis. Moreover, this approach was also found to be effective in other paper-based μPAD development. Thus, this work can be regarded as exceptional research towards commercialization.

### 6.4. CD–Nanocomposites for pH Dependence Discrimination of Biothiols

Towards enhanced analyte discrimination, the pH-tuned approach is effective as demonstrated by Au NP-based probes [130,131,132]. Xiang et al. proposed the use of a composite consisting of Ag NPs and nitrogen and sulfur co-doped CDs (N-, S-CDs; average size = 2.4 nm; λ_em_ = 425 nm at excitation of 350 nm) towards pH-dependent discriminative fluorescence “Turn-On” detection of biothiols [133]. Blue emissive N-, S-CDs were synthesized from the hydrothermal carbonization of citric acid monohydrate and L-cysteine. When compositing Ag NPs with N-, S-CDs, the PL emission of CDs was quenched due to the energy transfer process. When adding Cys, Hcy, and GSH to Ag NPs/N-, S-CDs at pH 3 (BR buffer), the energy transfer process was inhibited via a strong Ag–S bond, which led to the fluorescence “Turn-On” recovery. The linear ranges were established as 0.1–1000 µM for Cys, Hcy, and GSH with corresponding LODs of 68.5 nM, 82.6 nM, and 90.9 nM, respectively. On the other hand, only Cys and Hcy displayed the PL recovery at pH 7. This is because of the higher negative charge and steric effect of GSH at pH 7, which prevented it to penetrate through N-, S-CD shell layer to interact with Ag NPs surface. The linear regressions of Cys and Hcy-based PL recovery at pH 7 were 0.1–1000 µM. Note that both pH dependent sensory investigations showed the recovery of >100% in human serum samples and higher selectivity than that of other amino acids, GSSG, and sulfide ions. Thus, based on the attained results, this report can be regarded as exceptional research on a pH-tuned assay.

### 6.5. CD–Ag NP Nanocomposites for “Turn-On” Detection of Cys

Amjadi et al. synthesized the blue emissive CDs solvothermally (CDs; average size = 8 ± 2 nm; λ_em_ = 455 nm at excitation of 380 nm; PLQY = 11%) from pulp-free orange juice followed by compositing with Ag NPs (synthesized from sodium citrate and borohydride reduction) to afford a weakly emissive CD–Ag NPs system and employed them in “Turn-On” detection of Cys [134]. The formation of the CD–Ag NPs composite resulted to quenched emission due to the FRET mechanism. PL emission was restored by adding Cys to disrupt the CD–Ag NPs composite. The linear range of Cys was established as 6–300 nM with a LOD of 4 nM. The plasma and urine sample analysis on Cys detection displayed a recovery of >95%. This report claimed the high selectivity with negligible interference effects without providing experimental evidence. Thus, it can only be accounted as a preliminary study and requires further attention.

### 6.6. CD–AgOH Colloid for Discrimination between Cys and GSH

Zhou et al. synthesized the blue emissive CDs (CDs; average size = 4.6 nm; λ_em_ = 458 nm at excitation of 381 nm) from citric acid, ethylenediamine, and 1,3-propylenediamine by the hydrothermal method followed by compositing with an AgOH colloid for detecting Cys and GSH [135]. The AgOH colloid was composited by adding AgNO_3_ to the solution containing CDs at pH 6 (PBS buffer). When adding Cys to the AgOH colloid, it induced a shift in absorbance from 250 nm to 400 nm. However, the shift in absorbance was not observed when adding GSH. The selectivity study of the CD–AgOH colloid system subjected to metal ions showed greater PL quenching to Hg^2+^ and PL recovery to Cys and GSH. In order to distinguish between Cys and GSH, direct interactions of Cys and GSH with CD–AgOH colloids were proposed, which deliver unique “Turn-Off” and “Turn-On” responses via the inner filter effect, respectively. The linear quenching concentration of Cys was between 33 and 317 μM with a LOD of 3.17 µM. Likewise, the linear PL enhancement with GSH was between 16.7 and 100 μM with a LOD of 3.6 µM. The selectivity of Cys and GSH was high without the inclusion of Hcy. The GSH detection was also demonstrated by FBS recovery (>95%). However, further attention is required to attest its uniqueness.

### 6.7. CD–Au NPs for Fluorescent and Colorimetric Detection of Biothiols

Fu et al. developed the blue emissive nitrogen-doped CDs (CQDs; average size = 5 nm; λ_em_ = 438 nm at excitation of 360 nm; PLQY = 9.8%) from sucrose and glycine by the one-pot hydrothermal method followed by compositing with citrate capped Au NPs for colorimetric and fluorometric detection of biothiols [136]. When the CQD–Au NPs (final concentration of Au NPs was fixed at 1–5 nM) composite was formed, the fluorescence was quenched due to nanometal surface energy transfer (NSET) with a red color. When adding biothiols to the above composite, the NSET process was disrupted and led to fluorescent enhancement accompanied with a color change from red to purple via a strong Au–S bond. The linear PL and colorimetric regressions Cys were established as 0.05–12 µM and 0–100 µM, respectively, with a LOD of 20 nM (by both titrations). Moreover, this dual readout was effective at pH 7 (PBS). Similarly, Cys, Hcy, and GSH also displayed the dual readout response with exceptional selectivity to that of other amino acids. The human urine-based recovery of Cys was >98% with RSDs of <4.5%. Based on the unique NSET approach and dual readout responsivity, this work can be regarded as a nice innovation.

**Figure 11:**
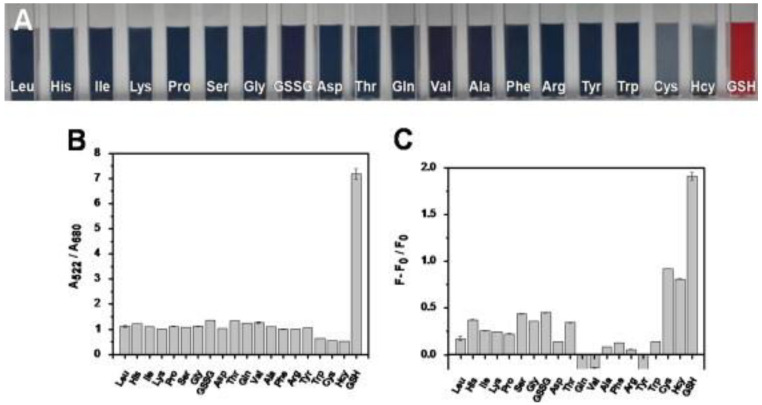
(**A**) Photographic images of solution containing Au NPs, CQDs, and various interferent molecules. (**B**) Absorption ratio A_522_/A_660_ of solution containing Au NPs, CQDs, and various interferent molecules. (**C**) Fluorescence recovery of solution containing Au NPs, CQDs, and various interferent molecules, where F_0_ represents the fluorescence intensity of the mixture of Au NPs and CQDs, and F is the fluorescence intensity of the mixture plus inspected species ((**A**–**C**) is reproduced with the permission from Ref. [137]).

To discriminate GSH against Cys and Hcy, dual readout nanosensors were demonstrated with carbon quantum dots and gold nanoparticle composites, as detailed in the following. Shi et al. synthesized the blue emissive CDs (CQDs; average size = 5 nm; λ_em_ = 460 nm at excitation of 370 nm; PLQY = 5.1%) from citric acid and 2,2′-(ethylene-dioxy) bis(ethylamine) by the microwave method followed by compositing with Au NPs (size = 12.8 nm; concentration = 4.37 nM) for superior dual readout detection of GSH against Cys and Hcy [137]. In the CQD–Au NPs composite, the FRET enabled fluorescence quenching occurred accompanied with a colorimetric change from red to blue and aggregation of nanoparticles. When adding GSH to the above system, the PL/colorimetric recovered due to the multi-dentate anchoring effect and specific steric structure of GSH, which stabilized the Au NPs (visually dispersed nanoparticles) via a strong Au–S bond and disrupted FRET to release the CQDs. The linear colorimetric and PL responses of GSH were established as 1–4 µM and 0.1–0.6 µM with a calculated LOD of 50 nM (by both titrations). The superior colorimetric/PL selectivity of GSH against Cys and Hcy is shown in Figure 11. This dual read-out of GSH in human plasma displayed remarkable recoveries of >80% with RSDs of <8%, thus it can be regarded as distinguished research towards GSH quantification.

Li et al. fabricated the blue emissive nitrogen and sulfur co-doped CDs (N-, S-CDs; average size = 2.5 nm; λ_em_ = 437 nm at excitation of 355 nm; PLQY = 77.2%) from citric acid monohydrate, and L-cysteine or L-serine by the hydrothermal method followed by compositing with Au NPs (average size = 12.9 ± 0.5 nm; concentration = 15.5 nM) for discriminating GSH [138]. PL emission was initially quenched due to FRET between N-, S-CDs and Au NPs and it then restored in the presence of GSH via strong Au–S bond-tuned energy transfer restriction. The superior GSH selectivity against Cys and Hcy was because of the multidentate anchoring effect and steric structural features. The linear regression of GSH with the N-, S-CD–Au NPs system was 0.01–5 µM with a LOD of 3.6 nM. On the other hand, the GSH linearity with N-CD–Au NPs was 0.1–5 µM with a LOD of 43 nM. Note that the GSH detection in human serum sample analysis showed recoveries of >93% with RSDs of <5.5%, thus it can be regarded as innovative research.

### 6.8. CD–MnO_2_ Nanocomposites for Selective Fluorescent Assay of GSH

The use of the CD–MnO_2_ system towards the FRET-based composite construction for discriminative quantification of GSH was proposed by researchers, as described next. Cai et al. synthesized a blue emissive CDs hydrothermally (CDs; average size ≤ 5 nm; λ_em_ = 435 nm at excitation of 360 nm) from citric acid and ethylenediamine followed by compositing with MnO_2_ nanosheets (obtained from KMnO_4_ source) for selective detection of GSH [139]. PL intensity at 435 nm was initially quenched due to the FRET between MnO_2_ and CDs, which was then restored with GSH. This is because that the GSH disrupted the FRET system via a strong Mn–S bond and delivered a “Turn-On” PL response. The linear range of GSH was 1–10 µM with a LOD of 300 nM and showed recoveries of >94% in spiked human serum samples with RSDs of <4%. The GSH selectivity at pH 7.2 (10 mM, Tris-HCl) was superior to that of all other competing species. However, the GSH selectivity by the CD–MnO_2_ system was still not justified in the presence of Cys and Hcy. Thereafter, Wang and co-workers proposed the use of CD incorporated MnO_2_ nanoflowers, as described below.

The blue emissive CDs (CDs; average size = 2.1 ± 0.25 nm; λ_em_ = 465 nm at excitation of 380 nm) were synthesized by the hydrothermal method from L-tryptophan followed by compositing with MnO_2_ nanoflowers (obtained from KMnO_4_ source) to afford CD–MnO_2_ NFs, which were applied in a discriminative assay of GSH against Cys and Hcy [140]. Fluorescence was initially quenched due to the FRET between CDs and MnO_2_ NFs. When adding GSH, PL intensity was recovered via the redox fascinated GSSG formation. The MnO_2_ oxidized the GSH to form GSSG and Mn^2+^ species to release CDs and to produce the “Turn-On” emission. On the contrary, Cys and Hcy underwent initial oxidation to produce stable sulfinate formation and led to a PL “Turn-Off” response. The linear GSH detection range was established as 2–200 µM with a LOD of 0.558 µM. The GSH selectivity was superior to that of all interfering species and was also applied in PC12 cellular (a type of catecholamine cells; cancer cell line) imaging studies. The best results on GSH detection were achieved at pH 5.5 within 5 min. Thus, this work can be stated as exceptional based on the attained results in the GSH assay. However, this work is a follow-up to the earlier report by Xu et al. [141], wherein, the blue emissive CDs, ^13^CDs and yCDs, were synthesized hydrothermally from citric acid and ethylenediamine, ^13^C-glucose and monoethanolamine, and glucose and phosphoric acid, respectively. The as-synthesized CDs were passivated with polyethylenimine (PEI) to afford pCDs (pCDs; average size = 4.6 nm; λ_em_ = 470 nm) followed by compositing with MnO_2_ to afford the pCD–MnO_2_ FRET system to be applied in the selective detection of GSH against Cys and Hcy by fluorometric and magnetic bimodal responses. The GSH was oxidized by MnO_2_ to afford GSSG and to restore the “Turn-On” emission of pCDs and magnetic response of ^13^CDs. The linear ranges of GSH by PL and magnetic responses were 1–200 μM and 5–200 μM with calculated LODs of 0.6 µM and 2.8 µM, correspondingly. Moreover, the selectivity of GSH was superior to that of all the analytes and was effective in magnetic resonance (MR) imaging studies. Based on the above results, this work can be regarded as unique and primary research.

### 6.9. Organic Moiety Conjugated CDs for a Selective Fluorescent Assay of GSH

The reaction-based “Turn-On” fluorometric detection of GSH was also proposed by researchers, as detailed next. Kong et al. synthesized the blue emissive CDs (CNDs; average size = 3.9 ± 0.5 nm; λ_em_ = 435 nm at excitation of 340 nm; PLQY = 9.9%) from citric acid and 4,7,10-trioxa-1,13-tridecane-diamine by the modified hydrothermal-solvothermal treatment in a poly(tetrafluoroethylene) autoclave [142]. The CNDs reacted with dopamine (DA) to afford a composite type CND–DA (a dopamine-quinone system) with corresponding fluorescent quenching, which can be restored in the presence of GSH via the reduction of dopamine-quinone. The linear PL recovery of GSH was 30–400 µM with a calculated LOD of 0.46 µM. The as-synthesized CNDs showed greater biocompatibility and were engaged in HUVEC cell (HUVEC = human umbilical vein endothelial cells) imaging studies. The GSH assay showed recoveries of >97% in human serum samples with RSDs of <6.5%. Although the GSH selectivity seems to be superior, this work requires further attention on interference studies with Hcy.

Yan et al. demonstrated the use of CDs-bromoacetyl bromide conjugates for a reaction-mediated fluorescence “Turn-On” assay of GSH against Cys and Hcy [143]. The blue emissive CDs (CDs; average size = 3.5 nm) were synthesized by the one-pot hydrothermal method from citric acid and diethylenetriamine (DETA) followed by functionalizing with bromoacetyl bromide to afford weakly emissive CD–Br (a composite model; average size = 5.5 nm; λ_em_ = 470 nm at excitation of 390 nm). The CD–Br only reacted with GSH via a specific reaction and displayed a discriminative “Turn-On” fluorescence response. The linear regression of GSH was 0–34 µM with a calculated LOD of 0.14 µM.

**Figure 12:**
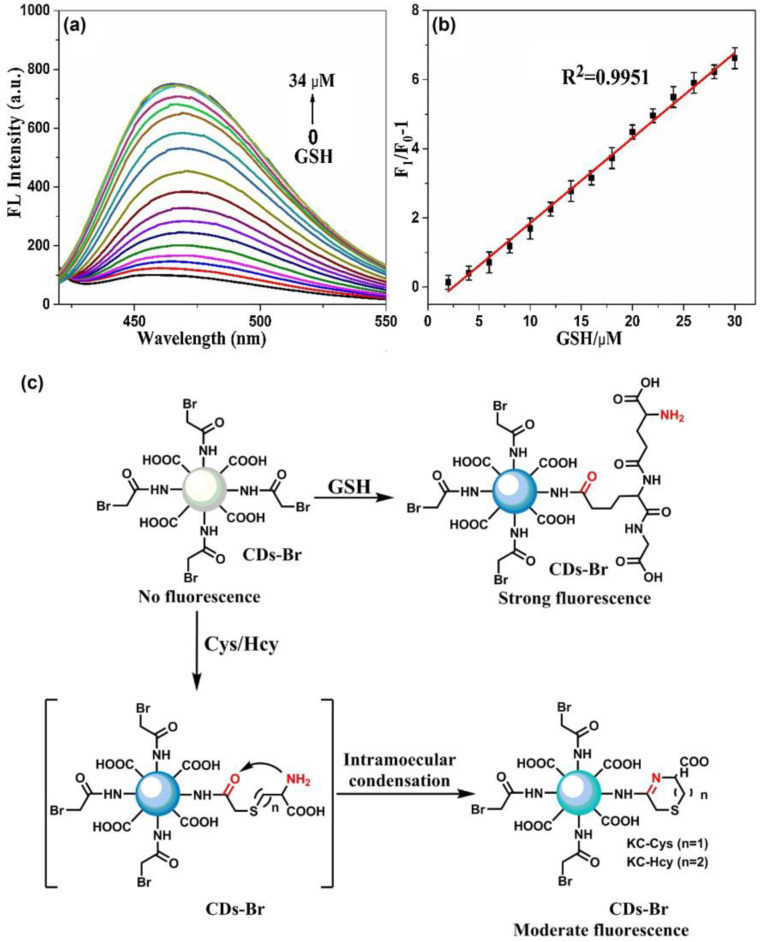
(**a**) The fluorescence spectra of CD–Br (0.5 mg/mL) upon the addition of different concentration of GSH (0.002 mol/L). (**b**) The plot of fluorescence intensity ratios of F_1_/F_0_ − 1 at 470 nm versus the concentration of GSH 0.01 mol/L PBS (pH = 8.0) at room temperature. (**c**) Proposed reaction for the turn-on fluorescent response of CD–Br to GSH against Cys/Hcy ((**a**–**c**) is reproduced with the permission from Ref. [143]).

The best fluorescence response was obtained at pH 8 (10 mM PBS buffer) after 40 min. This reaction-based GSH assay was effective over a wide range of interferences and also was demonstrated in HUVEC cellular imaging studies. Moreover, the GSH selectivity was operative over a wide range of temperatures between 25 °C and 60 °C. Figure 12 displays the linear PL enhancement of CD–Br in the presence of GSH and the unique reaction paths between Cys, Hcy, and GSH. Based on the observed results, this work can be regarded as innovative towards real-time monitoring of GSH against Cys and Hcy.

### 6.10. Tyr–CDs for Enzyme-Mediated Fluorescent Detection of Biothiols

The enzyme-mediated fluorescent “Turn-On” detection of Cys, Hcy, and GSH was proposed by using blue emissive L-tyrosine methyl ester capped CDs (Tyr-CDs; average size = 2.2 nm; λ_em_ = 455 nm at excitation of 350 nm; PLQY = 12.9%) [144]. Tyr–CDs were synthesized by directly pyrolyzing of citric acid followed by functionalizing with L-tyrosine methyl ester. The fluorescence was quenched via the generation of quinone when the L-tyrosine was mixed with tyrosinase enzyme (contains Cu^2+^). When adding biothiols to the above system, the quinone generation was inhibited via interaction with Cu^2+^ (Cu–S bond) in the enzyme, thereby leading to fluorescence recovery. The linear regressions of Cys, Hcy, and GSH were established as 1–50 µM, 5–50 µM, and 0.1–50 µM with estimated LODs of 0.12 µM, 3.5 µM, and 31 nM, respectively. The human plasma sample-based recoveries of Tyr-CDs to biothiols were from 90.80% to 118.50% with RSDs of <4%. Currently, this is the only report available on the enzyme triggered biothiols assay, thereby it can be regarded as innovative.

### 6.11. CD–DTNB System for Fluorescent Recognition of Biothiols

The inner filter effect triggered fluorescent “Turn-Off” detection of biothiols was pronounced by nitrogen-doped CDs as discussed below. Wu et al. synthesized the green emissive CD (N-doped CDs; size = 3–5 nm; λ_em_ = 510 nm at excitation of 410 nm; PLQY = 31%) from p-hydroxybenzoic acid and ethylenediamine by the hydrothermal method for detecting GSH in the presence of 5,5′-dithiobis-(2-nitrobenzoic acid) (DTNB) [145]. The N-doped CDs was used as the IFE fluorophore and the DTNB was engaged as the recognition molecule for biothiols. The N-doped CD–DTNB system (a composite model) alone did not show any fluorescence quenching. When adding GSH (at pH 7.4; phosphate buffer) to the above system, emission quenching occurred via a reaction between GSH and DTNB to afford TNB. Absorbance of TNB and N-doped CDs overlapped to induce the IFE-based fluorescent quenching. The linear range of GSH was 0.2–1000 µM with a LOD of 30 nM. A similar sensory response was attained by Cys and Hcy, with no information on the linear range and LODs. The work demonstrated by an oxidative stress model and SMMC-7721 cells (hepatocellular carcinoma cell line) showed high selectivity.

Yang et al. proposed the use of green emissive nitrogen-doped CDs (NCQDs; average size = 3.1 nm; λ_em_ = 512 nm at 409 nm excitation of 409 nm; PLQY = 29%) synthesized from catechol and ethanediamine by the hydrothermal method towards fluorescent “Turn-Off” detection of Cys, Hcy, and GSH at pH 7 (Tris-HCl) with a similar mechanistic approach [146]. The linear ranges of Cys, Hcy, and GSH to the NCQD–DTNB system (a composite model) were established at 1 nM–40 µM, 10 nM–40 µM, and 5 nM–40 µM with calculated LODs of 1.01 nM, 3.29 nM, and 3.23 nM, correspondingly. The biothiols selectivity by the NCQD–DTNB system was high with recoveries of >98% and RSDs of <4.5% in human serum samples; therefore, this IFE-based fluorescent assay of biothiols is much appreciated.

### 6.12. Cobalt-Doped CDs for Reaction-Tuned Fluorescent Detection of Cys

**Figure 13:**
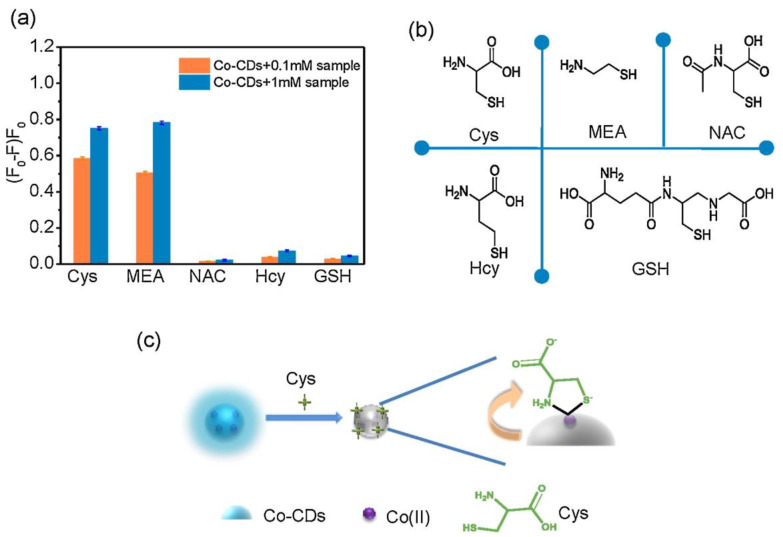
(**a**) Fluorescence response of Co–CDs upon addition of various concentrations of Cys, MEA, NAC, Hcy, and GSH. Data represents the average of three independent experiments done on different days. (**b**) Chemical structure of Cys, MEA, NAC, Hcy, and GSH. (**c**) Schematic recognition of Co–CDs to Cys ((**a**–**c**) is reproduced with the permission from Ref. [147]).

Liu et al. synthesized the blue emissive cobalt doped CDs (Co-CDs; average size = 3.46 nm; λ_em_ = 445 nm at excitation of 365 nm; PLQY = 44.6%) from folic acid and cobalt chloride by the hydrothermal method and utilized them in selective fluorescent “Turn-Off” assay of Cys against Hcy and GSH [147]. When adding Cys to Co–CDs at pH 7.4 (10 mM PBS), PL intensity was quenched linearly from 0.1 to 100 μM with a LOD of 80 nM. None of the interferences, including Hcy, GSH, and N-acetyl-L-cysteine (NAC), showed the similar static quenching response except the 2-aminoethanethiol (MEA). This might be due to the five membered chelate ring formation between Co in Co–CDs and -NH_2_ and -SH of Cys and MEA, as shown in Figure 13. In human serum samples, the Co–CDs to Cys showed recoveries of >98% with RSDs of <7%. This is the only report of a Cys assay with a fluorescent “Turn-Off” tactic, thereby it can be regarded as remarkable research. Similar to the fluorescent tactics, CDs-enabled peroxide mimics by the oxidation of 3,3,5,5-tetramethylbenzidine (TMB) in the presence of H_2_O_2_ were also reported in a colorimetric assay of biothiols [148,149], which also approved the utility of CDs in Cys, Hcy, and GSH assays.

## 7. Probe/CDs Selection and Sensory Requirements

The development of fluorescent CDs-based probes towards selective detection and quantification of Cys, Hcy, and GSH must fulfil certain requirements, as illustrated below.

1. The uniqueness of a CDs-based fluorescent assay of biothiols depends on the size and PLQY. Therefore, to obtain CDs with a proper size and PLQY, it is essential to identify the precursor reactants and suitable synthetic tactics.
2. To attain high biothiols selectivity in CD–metal complex-mediated detection, the CDs must possess specific selectivity to metal ions with thiophilicity nature (such as Hg^2+^, Ag^+^, Cu^2+^, Fe^3+^, Au^3+^, etc.). Therefore, to be able to interact with those metal ions, CDs must possess functional units, such as -NH_2_ and -COOH, or be doped with N, S, P, etc. However, in the case of doping, the concentration must be carefully tuned to achieve the expected results.
3. Dye-incorporated CDs towards consecutive ratiometric discrimination of metal ions and biothiols depends on the precise concentration of dye molecules. Thus, it is essential to optimize the dye concentration before designing such innovative probes.
4. To attain greater sensory responses to biothiols using the CDs incorporated in composites, it is necessary to choose compositing material involved in the detection process/mechanism with thiophilicity.
5. For dual readout fluorescence and colorimetric detection of biothiols, the CDs must be composited with the colorimetric probe, such as Au NPs. The composition ratio must be fixed to achieve significant results.
6. Reaction-based sensory responses of CDs to biothiols depend on the reacting units functionalized over the carbon dot surface. Thus, it is necessary to identify molecules to be functionalized over the CD surface at required concentrations that are highly reactive to specific biothiols.
7. It is essential to categorize the exact mechanisms of the selective sensing of biothiols with CDs-based probes. The coordinative bindings and mechanistic approaches, such as PET, FRET, IFE, and NSET, must be clarified for the emerging new designs.
8. To commercialize the CDs-based biothiols assay, the exact pH conditions with given details on buffer solutions and concentrations, incubation time, operative temperature, and interference effect must be clarified for researchers.

## 8. Advantages

CDs-based fluorescent probes and their utilization in biothiols assays have the following advantages, as stated below.

1. Around 75–80% of reported CDs were synthesized using the one-pot reaction with high PLQY, which is comparable to the current nanoprobes engaged in the detection of different analytes [150,151,152].
2. CDs-based fluorescent assays of biothiols are equal with inorganic, organic, and hybrid nanoprobes in low toxicity and biocompatibility when the analyte detection is conducted in an aqueous environment [153,154,155].
3. As discussed in earlier sections, the performance of the CD–metal complex-mediated biothiols detection and quantification is comparable to many metal complex-mediated sensors [156,157,158], which is worthy of attention.
4. Incorporation of dye molecules and compositing Au NPs with CDs may enhance the ratiometric and colorimetric response towards biothiols. The CDs-based designs are as inspiring as those dye-based sensors [159,160,161].
5. CDs hold the promise as potentially safe vehicles for biological sample-based biothiols assays due to low in toxicity and high biocompatibility. Moreover, toxicity of CDs can be further reduced by compositing with low toxic nanomaterials, such as Ag NPs, Au NPs, and nanoclusters, to engage in bioimaging and therapeutic applications.
6. CDs-nanocomposites are comprised of highly selective reactive species (in the presence of biothiols), which can avoid the interference effect. Likewise, CDs are also able to discriminate Cys, Hcy, and GSH via tuning of the pH environment.
7. Construction of the red-green-blue (RGB) emitting CDs-nanocomposites is possible by mixing CDs with red to blue emissive nanomaterials, which can be utilized for biothiols assay over a broad PL range.
8. CDs-based fluorometric discrimination of biothiols can be effectively applied in real samples, such as human serum, FBS, plasma, urine, etc. This can be noted as a great advantage towards the development of a unique analytical method.

## 9. Limitations

CDs-based fluorometric biothiols detection and quantification also have the following limitations, as mentioned below.

1. Development of fluorescent CDs with high PLQY are limited by the use of precursors and reaction conditions [162,163]. The sensory investigations require careful optimizations of the pH, incubation time, operative temperature, etc.; therefore, real-time detection can be time consuming.
2. In CD–metal ion pair-based biothiols assays, the formation of metal complexes, such as CD–Hg^2+^, CD–Ag^2+^, and CD–Cu^2+^, may increase toxicity. Hence, the use of such complex-mediated biothiols assays may harm the biological environment or cell lines, which should be carefully examined.
3. In general, CDs-based specific sensory responses to biothiols are limited by the functional units or doped elements. Thus, careful optimization is mandatory to ensure the existing functional units or doped elements are at required concentrations.
4. Dye molecules combined with CDs for ratiometric sequential detection of metal ions and biothiols is limited by the concentration and overlapping efficacy of dye molecule, which requires great attention.
5. CDs-nanocomposites formation for FRET/IFE-based biothiols assays is limited by the composition ratio of CDs and composting material. Otherwise, the primary quenching by FRET or IFE can affected significantly. In case of IFE, it is also essential to clarify the absorbance overlapping of the compositing materials.
6. The reaction-based biothiols assay is limited by the solid evidence of the mechanistic pathway. In such cases, a model reaction must be conducted to support the proposed mechanism.
7. Characterization of CDs and detailed mechanistic studies on CDs-based biothiols assay require instruments such as high-resolution transmission electron microscopy (HRTEM), dynamic light scattering (DLS) analyzer, X-ray photoelectron spectroscopy (XPS), fourier transform infrared (FTIR) spectroscopy, X-ray diffraction (XRD), etc. Thus, CDs-based biothiols discrimination is limited by the available instruments and cost-effectiveness.
8. In many reports, CDs-based biothiols quantification was not demonstrated by an interference effect. and discrimination between Cys, Hcy, and GSH was unavailable. Therefore, real sample-based recoveries and bioimaging remain a concern.

## 10. Conclusions and Perspectives

In this review, fluorescent CDs-based biothiols detection and quantification are discussed in detail. The synthetic methods involved in the fabrication of fluorescent CDs were clearly delivered. Thereafter, the use of (1) a CD–metal ion system and (2) CD–nanocomposites towards biothiols quantification were comprehensively illustrated with their real-time applications in real samples (such as human serum, plasma, and urine) and bioimaging studies. The uniqueness and deficiencies of each report was clearly stated and commented. Finally, the selection of CDs/sensory probe, sensory requirements, merits, and limitations were discussed for the readers. However, some perspective points must be given more attention, as noted below.

1. Many reports of CDs-based fluorescent detection of biothiols followed difficult procedures and did not provide reliable information on the interference effect, which should be rectified to be considered “state-of-the-art”.
2. Up until now, reports on green and red emissive CDs-based assays of biothiols are insufficient, which should be the focus for future research towards a wide range of applications.
3. Although research on using CD–metal ion pairs or composites for detecting Cys, Hcy, and GSH has become the mainstream, not much detail was given on how to distinguish among them. Because Cys, Hcy, and GSH are involved in many different biological processes, this issue should be addressed in the future.
4. In some reports, information regarding the PLQY, exact cause of CDs emission, and PL quenching type (static/dynamic) of CDs with metal ions and during composites formation was not clarified for the readers. These issues should be stated more clearly in future studies.
5. CD–Hg^2+^ metal complex-mediated fluorescent “Turn-On” sensing of biothiols via a strong Hg–S affinity was proposed in many reports, but none of them were commercialized into practical use [164,165]. This issue should be focused on in the future.
6. The majority of reports delivered recoveries of CD–Hg^2+^ complex-mediated biothiols assays in biological samples (such as human serum, plasma, urine, etc.) without giving information on the toxicity of the CD–Hg^2+^ metal complex, which should be clearly addressed in the future.
7. So far, only Hg^2+^, Ag^+^, Cu^2+^, Fe^3+^, and Au^3+^ were reported in CD–metal ion pair enabled biothiols assays based on the thiophilicity of metal ions. This approach should be expanded with other thiophilic metal ions, such as Pb^2+^, Cd^2+^, Mo^4+^, etc.
8. Reports on dye molecules incorporated in the CD–metal ion pair system for ratiometric detection of biothiols are still insufficient. Future research should focus on using other dye molecules and justifying the role of dye molecules.
9. The CDs–nanocomposites system for FRET-tuned PL “Turn-On” detection of biothiols can be improved by encouraging more research.
10. To date, only one report is available on the reaction-based PL “Turn-On” dual channel discrimination between Cys, Hcy, and GSH [128], which should be expanded with other biothiols reactive species.
11. Reports on the fabrication of microfluidic paper-based analytical devices from vinyl sulfone clicked CDs for fluorescent assays of biothiols were impressive and could be commercialized. Thus, a similar approach should be strongly encouraged.
12. Only one report is available so far on CD–nanocomposite (Ag NPs/N, S-CDs)-based pH dependence discrimination between Cys, Hcy, and GSH [133], which should be a future research focus.
13. Au NPs and CDs composites displayed dual readout (fluorescent and colorimetric) responses to a specific analyte GSH against Cys and Hcy, which requires more attention in future research.
14. The CD–MnO_2_ composite system and CD–Br system selectively detects the GSH against Cys and Hcy via redox or specific reactions, thereby such approach can be anticipated for biological applications and towards commercialization.
15. CD–DTNB and Co–CDs (metal doped CDs) composite models showed IFE and reaction-tuned direct recognition of biothiols and Cys, respectively, via the PL “Turn-Off” response against Hcy and GSH. This approach must be improved by more similar research.
16. Reports on CDs-based discrimination of Cys against Hcy and GSH, and GSH against Cys and Hcy are available. However, there is no report on the discrimination of Hcy against Cys and Hcy, which should be the focus towards groundbreaking achievements.
17. The emission of CDs can be enhanced by combining a surface plasmon-coupled emission (SPCE) platform and photonic crystal-coupled emission (PCCE) technology for distinct detection of biothiols at a lower concentration (<nM).
18. Until now, CDs-based fluorescent assays of biothiols lack theoretical support, which should be addressed by density functional theory (DFT) investigations in the future [166,167].

Currently, many research groups are working on developing new CDs-based sensory probes to rectify the aforementioned issues. In terms of PL “On” or “Off” responses to biothiols with real-time applicability, research on CDs-based biothiols assay tactics can be noted as exceptional with great anticipation and excitement.

## Figures and Tables

**Table 1:** Difference between Top-down and Bottom-up approaches in CDs synthesis.

Comparison Parameters	Top-Down Approach	Bottom-Up Approach
Basic principle	Sequential cutting or grinding of bulk material into CDs	Synthesis of CDs from smaller atom or molecules
Carbon source	Solid state materials are used as source	Gaseous or liquid materials are engaged as source
Processing method	Physical method	Physical and chemical methods
Advantages	(1) Can produce CDs at a large scale (2) Deposition of CDs over a large substrate can be done (3) Purification of CDs is not required	(1) Can synthesize ultra-small particles (2) Parameters can be controlled during deposition (3) Cost-effective and cheaper method (4) Can easily operate in an eco-friendly manner
Limitations	(1) Produce CDs with varied particle shapes and a broad size distribution (2) Synthesis involves complex steps and harsh conditions (3) Controlling deposition parameters is difficult (4) Expensive method	(1) Large scale production of CDs is difficult (2) Chemical purification of CDs is necessary
Structure and QY	Produce weakly emissive CDs (QY ≤ 10%) with a graphite-like structure	Produce highly emissive CDs (QY ≥ 10%) with an amorphous structure

**Table 2:** Linear ranges, LODs, suitable assay pH, and applications of fluorescent biothiols assay by CD–Hg^2+^ ion pair.

Emissive CDs (PLQY)	Synthetic Tactic	Proposed CD–Metal Ion Pair	Linear Range	Detection Limits (LODs)	Suitable Assay pH	Applications	Ref
Blue emissive CDs (11%)	Calcination	CD–Hg^2+^	0.01–5 μM (for Cys, Hcy, and GSH)	4.9 nM, 6.1 nM, and 8.5 nM, respectively	pH 8.5	Fetal bovine serum analysis	[91]
Green emissive CDs (12.3%)	Hydrothermal	CD–Hg^2+^	0.5–10 μM (for Cys, Hcy, and GSH)	80 nM, 76 nM, and 69 nM, respectively	pH 6.5	Human plasma analysis	[92]
Blue emissive CDs (56%)	Hydrothermal	CD–Hg^2+^	0–40 μM (for Cys, Hcy, and GSH)	110 nM, 110 nM, and 130 nM, respectively	pH 7.4	NA	[93]
Blue emissive CDs (81.94%)	Hydrothermal	CD–Hg^2+^	NA	NA	NA	NA	[94]
Blue emissive NSCDs (31.8%)	Pyrolysis	NSCD–Hg^2+^	1–10 µM, 0.2–2.5 µM, and 0.1–2.0 µM (for Cys, Hcy, and GSH, respectively)	23.6 nM, 12.3 nM, and 16.8 nM, respectively	pH 7.4	HeLa cellular imaging studies	[95]
Green emissive PCDs (63%)	Hydrothermal	PCD–Hg^2+^	1–45 µM, 0–15 µM, and 0–30 µM (for Cys, Hcy, and GSH, respectively)	60 nM, 20 nM, and 35 nM, respectively	pH 7	Human urine analysis	[96]
Red emissive BN-CDs (18%)	Hydrothermal	BN-CD–Hg^2+^	5–200 µM, 5–100 µM, and 5–225 µM (for Cys, Hcy, and GSH, respectively)	1.7 µM, 2.3 µM, and 3 µM, respectively	pH 7.4	HepG2 cellular imaging studies	[97]
Blue emissive CDs (14.3%)	Microwave method	CD–Hg^2+^	0.1–20 µM and 0.2–45 µM (for GSH and Cys)	30 nM and 50 nM (for GSH and Cys)	pH 6	NA	[98]
Blue emissive CNPs (30%)	Microwave-assisted hydrothermal method	CNP–Hg^2+^	1–6 µM (for Cys)	15 nM (for Cys)	pH 5–10	A549 cellular imaging studies	[99]
Blue emissive NCDs (35.4%)	Hydrothermal	NCD–Hg^2+^	0–50 μM (for Cys)	0.79 nM (for Cys)	pH 7	NA	[100]
Blue emissive NCNDs (34.5%)	Hydrothermal	NCND–Hg^2+^	1–10 µM (for Cys)	40 nM (for Cys)	pH 7	Human urine analysis	[101]
Blue emissive N-S-CDs (NA)	Microwave-assisted hydrothermal method	N-S-CD–Hg^2+^	5–50 µM (for Cys)	400 nM (for Cys)	pH 7	NA	[102]
Blue emissive CQDs (NA)	Microwave-assisted hydrothermal method	CQD–Hg^2+^	0.10–2.0 mU mL^−1^ (for GSH)	0.050 mU mL^−1^ (for GSH)	pH 6	Glutathione reductase activity study	[103]
Blue emissive N-CDs (40% ± 0.06)	Solid state method	N-CD–Hg^2+^	0–32 µM (for GSH)	40 nM (for GSH)	pH 7.4	BHK cellular imaging studies	[104]
Blue emissive NSCDs (NA)	Microwave method	NSCD–Hg^2+^	0.5–34 µM (for GSH)	52 nM (for GSH)	pH 7	Human urine, blood serum and HepG2 cellular imaging studies	[105]
Blue/Red ratiometric emissive NSCDs (15.5%)	Hydrothermal	NSCD–Hg^2+^	220–400 μM (for GSH)	15.7 nM (for GSH)	pH 7–8	in-vitro and in-vivo bioimaging studies	[106]

NA = Not available; µM = Micromole (10^−6^ M); nM = Nanomole (10^−9^ M); mU = milli Units; mL = milliliter.

## Data Availability

Not applicable.
